# Planar Covariation of Hindlimb and Forelimb Elevation Angles during Terrestrial and Aquatic Locomotion of Dogs

**DOI:** 10.1371/journal.pone.0133936

**Published:** 2015-07-28

**Authors:** Giovanna Catavitello, Yuri P. Ivanenko, Francesco Lacquaniti

**Affiliations:** 1 Centre of Space Bio-medicine, University of Rome Tor Vergata, 00133, Rome, Italy; 2 Laboratory of Neuromotor Physiology, Santa Lucia Foundation, 00179, Rome, Italy; 3 Department of Systems Medicine, University of Rome Tor Vergata, 00133, Rome, Italy; University of Portsmouth, UNITED KINGDOM

## Abstract

The rich repertoire of locomotor behaviors in quadrupedal animals requires flexible inter-limb and inter-segmental coordination. Here we studied the kinematic coordination of different gaits (walk, trot, gallop, and swim) of six dogs (*Canis lupus familiaris*) and, in particular, the planar covariation of limb segment elevation angles. The results showed significant variations in the relative duration of rearward limb movement, amplitude of angular motion, and inter-limb coordination, with gait patterns ranging from a lateral sequence of footfalls during walking to a diagonal sequence in swimming. Despite these differences, the planar law of inter-segmental coordination was maintained across different gaits in both forelimbs and hindlimbs. Notably, phase relationships and orientation of the covariation plane were highly limb specific, consistent with the functional differences in their neural control. Factor analysis of published muscle activity data also demonstrated differences in the characteristic timing of basic activation patterns of the forelimbs and hindlimbs. Overall, the results demonstrate that the planar covariation of inter-segmental coordination has emerged for both fore- and hindlimbs and all gaits, although in a limb-specific manner.

## Introduction

Quadrupeds have the ability to generate adaptive coordination and exhibit versatile gait patterns (walk, trot, pace, bound, gallop, etc.) in order to move at different speeds or under different environmental conditions. All gaits are either symmetrical or asymmetrical, and involve a lateral-sequence or a diagonal-sequence [1]. Several modes of legged terrestrial locomotion can be simplified in terms of two general mechanisms, a pendulum and a spring-mass system, which are utilized either separately or in combination [2–5]. In addition, for both terrestrial and aquatic locomotion, midline stabilization and maneuverability can be increased by controlling side-to-side, mutually opposing forces [6]. Dynamic changes in the gear ratio of muscles can also enhance the performance of skeletal muscles by maintaining them at the shortening velocities that maximize their power or efficiency in trotting and galloping [7].

Animals are complex, high dimensional, dynamical systems and one of the promising approaches consists in looking at the so-called templates and modular organization of their movements [8–12]. Thus, principal component analysis is a powerful method of data reduction aimed at obtaining low-dimensional approximation of high-dimensional processes [11,13–17]. Various methods have been used to model the organization of limb and muscle coordination during canine locomotion [12,18–31]. Earlier works also demonstrated that muscle mechanoreceptors and proprioceptive reflexes contribute to the phase-relationships and coordinated joint angular movements during dog locomotion [18,32–34]. However, the issue of a modular control of limb kinematics received little attention. Here we explored the framework of kinematic modules for understanding the dimensional complexity and control of dog locomotion under different environmental conditions.

The inter-segmental limb coordination typically shows adaptive behavior during different gaits [16,35]. In particular, a planar covariation of the temporal changes of limb segment elevation angles has been demonstrated during different gaits in a few animal species, including humans [14], Rhesus monkeys [9] and cats [36,37]. Studying how the coordination patterns change in different locomotion conditions can lead to a better understanding about how the central pattern generators (CPG) control the timing of inter-limb coordination. In this respect, aquatic locomotion may represent a distinctive condition for revealing the intrinsic properties of the inter- and intra-limb coordination, without the constraint of the terrain substrate for the limbs during stance. Evolutionary conservation of ancestral neural networks [38–41] involves both biomechanical and neurophysiological aspects of quadruped limb coordination. The inter-limb phase and the inter-segmental coordination pattern can be controlled by symmetrically organized unit burst generators for each joint, limb segment, or groups of muscles [42,43] and may emerge from the coupling of neural oscillators with limb mechanical oscillators [35,37]. Therefore, investigating both inter-limb and inter-segmental phase patterns may characterize phase relationships between neural oscillators and CPG organization in different gaits [44]. The application of principal component analysis and neural networks to myoelectric signal analysis may capture the general structure of neural control for generating rhythmical motor patterns [45–50]. Indeed, the dynamic behavior of the musculo-skeletal system can be modelled through a linear combination of a small number of basic muscle activation patterns that reflect the shaping function of CPG [51]. Muscle activations tend to intervene during limited time epochs [52] and the biomechanical correlates of each activation pattern have been described [53–55]. It is worth noting that the characteristics timing of muscle activation is gait dependent [56,57], although their inter-limb dependence received less attention [58].

In this study, we aimed at comparing the inter-segmental coordination pattern during various forms of quadrupedal locomotion. To this end, we recorded the kinematics of different gaits (walk, trot, gallop, and swim) of six dogs (*Canis lupus familiaris*), all of the same or closely related breeds (Golden or Labrador Retrievers) and similar size (weight ∼35 kg, height ∼57 cm at the withers). An important feature of our study is that the behavior of all the animals was observed outdoors in a naturalistic setting. Because we used a completely non-invasive (markerless) field-recording, some control of accuracy was inevitably sacrificed. However, we separately verified the results obtained with the field-recording with those obtained with a high-performance 3D motion-capture system, and found good agreement. We examined the inter-limb coupling and the inter-segmental coordination in forelimbs (FL) and hindlimbs (HL) and, in particular, the planar covariation of limb segment elevation angles using the model of the tri-segmental limb [9,14,22,37]. Finally, to investigate a characteristic timing of muscle activation and its potential link to the observed differences in the inter-segmental phase pattern of FL and HL, we used available published data on muscle activity during canine locomotion [59] and decomposed them into basic activation patterns using a factorization algorithm [60].

## Materials and Methods

### Ethics statement

No special permission is required in Italy for non-invasive observation of animals (here, the dogs) outside laboratory settings in behavioral studies like the present one (Italian Normative 26/2014). All recordings were made in collaboration with members of the “SICS, Scuola Italiana Cani Salvataggio Tirreno” during normal training procedures of dogs. Permission for video recording of dog behavior was obtained from dog owners who were briefed as to what type of gait would be encouraged for the dog and gave consent before the test could commence.

### Animals and protocols

We used six healthy domestic dogs (*Canis lupus familiaris*): three Golden Retrievers and three Labrador Retrievers (weight 35±4 kg [mean±SD], height at the withers 0.57±0.4 cm, see Table 1). All recordings were made in collaboration with members of the “SICS”, that provided the dogs. SICS had previously trained the selected dogs for companionship and rescue exercises, but it did not train them to use one or another specific gait. Consequently, all dogs were very compliant to the instructions during the experiments on ground and in water. In particular, we were able to instruct the dogs to perform each locomotion task in the required direction, i.e., roughly orthogonal to the direction of the recording video camera. For the terrestrial locomotion session, the dogs walked, trotted and galloped in a large horizontal open space at their preferred speed. Data collection took place outdoors, in areas where there was ample space for dogs to move. For the swimming session, the recordings were performed during a dog show, in which the dogs were swimming in a special pool (∼10 m × 5 m) with transparent walls that allowed the audience to see their movements under water. For galloping and swimming, the dogs ran and swam alone towards the handlers at the opposite end of the pathway (rescue dogs are trained to reach as fast as they can a person that is yelling for help). For the other gaits, the handlers walked or ran aside the dog without any leash restriction. For walk, the dogs followed the walking handlers after a vocal command specific for this gait. For trot, the command was similar, but the handlers encouraged the dog to maintain a trot gait. Specific gait in dogs is typically determined by speed [30] and its categorization (Table 1) was confirmed a posteriori: all dogs displayed a lateral sequence walk, a trot with synchronized diagonal limb movements and a transverse gallop with a forelimb-initiated aerial phase [59].

**Table 1:** Dog characteristics and the number of recorded strides.

Characteristics					Number of strides recorded, hindlimb				Number of strides recorded, forelimb			
dog	breed	age, yr	weight, kg	height^a^, m	walk	trot	gallop	swim	walk	trot	gallop	swim
A	Golden Retriever	10	30	0.54	6	1	21	4	6	0	19	3
G	Labrador Retriever	8	30	0.53	7	6	8	4	7	5	7	4
K	Labrador Retriever	5	40	0.60	2	15	8	4	3	14	4	4
M	Golden Retriever	3	38.5	0.60	4	8	8	4	4	8	6	4
R	Labrador Retriever	5	34	0.56	6	9	5	5	6	10	3	5
S	Golden Retriever	10	35	0.63	7	5	4	7	7	4	4	6

^a^ The height is measured at the withers during standing, and corresponds to the distance between the ridge of the scapular blades and the ground.

### Data recordings

**Fig 1:**
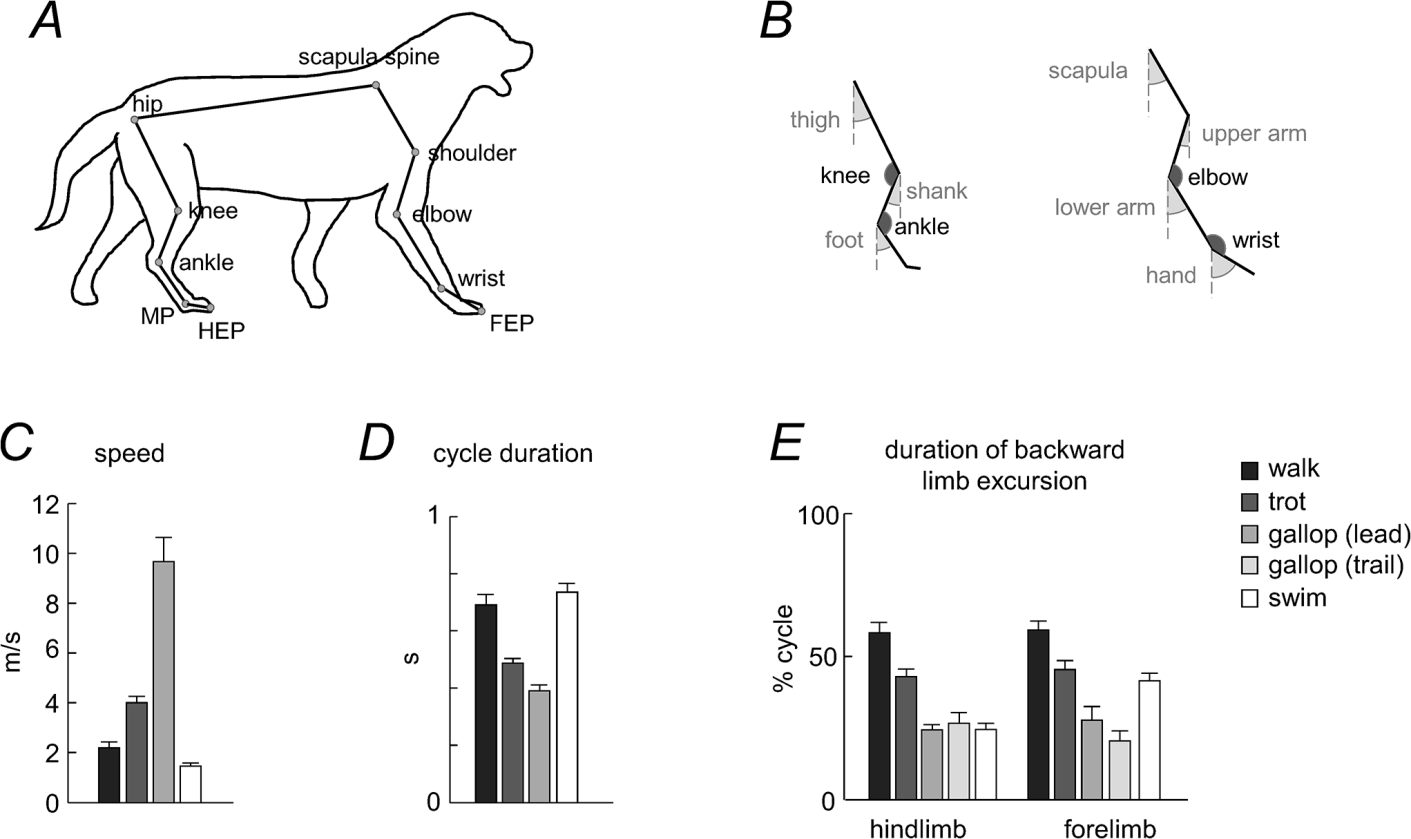
General gait parameters. A: schematic drawing of a walking dog, with the stick diagram representing the analyzed segments and markers. B: analyzed segments with joint and segment elevation angles. C: mean speed (+SD, n = 6). D: cycle duration. E: duration of backward (relative to the body) limb excursion. For galloping (asymmetrical gait), both leading and trailing limb values were computed.

The recordings were made using a Fujifilm Camera (FinePix SL1000, at 60 Hz) in order to obtain two-dimensional coordinates of selected landmarks (Fig 1A). The dogs moved in a direction roughly perpendicular to the optical axis of the recording camera to minimize errors in 2-D kinematic analysis [61]. The distance between the camera and the dog was about 8–10 m during terrestrial recordings and ∼5 m for aquatic locomotion, allowing us to record about 2–7 strides in each trial depending on the stride length (more strides were recorded during swimming due to slower motion). The recordings of locomotion were performed in both directions in order to analyze the kinematics of both the right and left side. For symmetrical gaits (walk, trot, swim), the kinematic data of the left and right limbs were pooled together because both sides have similar locomotion characteristics and functions. For the asymmetrical gait (gallop), we analyzed separately the gaits in which the limb was acting as either the trailing limb (i.e. the first limb to touch down) or the leading limb (i.e. the second limb to touch down). For two dogs, we failed to record the kinematics of the leading or trailing limb during gallop, as it did not face the camera.

### Data analysis

The types of gait were manually distinguished. We identified successful sequences of steps–those when the gait occurred in the dog’s sagittal plane steadily and on a straight path (starts and stops excluded) roughly perpendicular to the optical axis of the camera. On average, for each terrestrial gait we analyzed 14±7 successful strides per animal (247 strides total), and for swimming we analyzed 9±2 successful strides (54 strides total, Table 1). While the number of recorded strides differed somewhat between gaits/animals (Table 1), the inter-stride kinematic data for dog locomotion are known to be repeatable (e.g., [62]). Moreover, it is worth noting that a comparison between FL and HL segment planar covariation was performed at the same speed and on about the same number of strides. Finally, we provided information about the orientation of the covariation plane for individual strides (see *Results*).

Once we obtained the video, the reconstruction was performed using the software Tracker (v.4.85), a video analysis and modeling tool built on the Open Source Physics Java framework. The anatomic landmarks of the ipsilateral side (with respect to the camera) tracked in the reconstruction were: hip joint, knee joint, ankle joint, metatarsophalangeal joint (MP), endpoint (digital tip) of the hindlimb (HEP), scapular fulcrum, shoulder joint, elbow joint, wrist joint, and endpoint of the forelimb (FEP). These landmarks were used for the kinematic analysis and assessment of the inter-segmental coordination in the fore- and hindlimbs. Tracking of scapula during swimming was sometimes problematic (since the scapula was in the close vicinity of the surface of water) Nevertheless, in all analyzed strides, its waveform showed similar back-and-forth angular oscillations. To assess the diagonality of gaits (the sequence of fore- and hind-footfalls), the endpoints of the contralateral forelimb and hindlimb were also tracked. The trunk length of each dog (scapula-hip, Fig 1A) was measured prior to the experiments and was used as a metric scale to convert the 2-D video coordinates into real-world 2-D coordinates. Since the trunk length changes during locomotion (especially during galloping), we computed its mean length across each trial and used it for scaling under the assumption that it corresponds to that during quiet standing. While there might be a small discrepancy between the mean trunk length during locomotion and standing, this may only slightly influence the estimated speed (Fig 1C). Importantly, the estimates of angular movements of the limb segments and the principal component analysis are not affected by trunk length changes.

We first low-pass filtered (10 Hz, fourth-order dual-pass Butterworth) the kinematic data, and then we used a model-based algorithm that optimizes the locations of joint centers by constraining changes in the limb segment lengths (similar to the concept of a template-skeleton for accurate tracking with markerless motion capture systems, [63]. As a template, we used the average segment lengths calculated over all frames of the trial. The average accuracy of kinematic reconstruction, assessed as the mean coefficient of variation of the limb segment lengths during recorded locomotion, was 0.0012 ± 0.0011% (pooling together the data for all segments and gaits). We will describe further checks on the accuracy of kinematic analysis in the section “Potential inaccuracies of the markerless procedure for segment motion reconstruction.”.

### General gait parameters

The gait cycle for each limb was defined as the time-interval between two successive maxima of the horizontal motion of the distal point of the respective limb (relative to the most proximal marker of the limb). Speed was calculated from the distance the dog (hip landmark) covered during the stride. Duration of rearward (relative to the body) limb movements (that is the stance phase during terrestrial locomotion and the power stroke during swimming) was calculated for each limb. Limb endpoint excursion was determined separately for fore-aft and up-down (relative to the body) movements. To compare different dogs, the calculated values were normalized to hindlimb length (L). To compare the waveforms of limb segment motion across gaits (walk, trot, gallop and swim), we time-normalized (stretched) the data of all gaits to the same relative stance duration as that of walking. To this end, all gaits were scaled to 100 points originally and then scaled/normalized again to the same stance duration as walking (58% gait cycle) using the ‘interp1.m’ function of Matlab (so that all strides were again finally scaled to 100 points).

### Inter-limb coupling

To evaluate the inter-limb coupling, the phase lag (*PL*) between limbs was determined using methods previously described [1,64–66]. In brief, the relative timing of limb cycle onset was expressed as a percentage of the gait cycle:

$$PL=\frac{\Delta t_{i}}{T}\times 100\%$$ (1)

where Δ*t*_*i*_ is the interval of time between the cycle onset of the hindlimb ipsilateral relative to the recording camera and the cycle onset of the *i* ^*th*^ limb (*i* = 2,3,4), and *T* is the cycle duration of the hindlimb. According to this method, lateral sequence footfalls (ipsilateral fore/hind limb cycle onsets at similar instances) are determined at a value of 0%, while diagonal sequence footfalls (contralateral fore/hind limb cycle onsets at similar instances) are determined at a value of 50%. Intermediate values (∼25%) correspond to no limb pairing. For galloping, *PL* was calculated with respect to the limb ipsilateral to the recording camera and acting as the trailing limb.

### The tri-segmented limb

The model of the dog limbs consisted in an interconnected chain of rigid segments (Fig 1A), following the tri-segmental limb scheme of mammals [67]. The analyzed segments were: trunk (scapula spine-hip), thigh (hip-knee), shank (knee-ankle), foot (ankle-MP), scapula (scapula spine-shoulder), upper arm (shoulder-elbow), lower arm (elbow-wrist) and hand (wrist-FEP, including toes). The elevation angle of each segment in the sagittal plane corresponds to the angle between the projected segment and the vertical (Fig 1B). The angles are positive when the distal marker is located anterior to the proximal marker. Movements of the toes in the hindlimb contribute relatively little to the kinematics of forward progression and this distal part of the hindlimb is relatively short. If we omit these most distal segments, the hindlimb is functionally tri-segmented [67]. Therefore, from this functional perspective, we used the serially homologous forelimb segments for comparing fore- and hindlimbs: upper arm—thigh, lower arm—shank, and hand–foot (Fig 1B). Nevertheless, the scapula also undergoes significant rotations in the sagittal plane in most mammalian groups during locomotion. Therefore, in a complementary analysis of the inter-segmental coordination we included a proximal segment (scapula) in the serial tri-segmental forelimb model [67]: scapula—upper arm—lower arm (Fig 1B).

### Potential inaccuracies of the markerless procedure for segment motion reconstruction

Even though the Tracker software allowed a reliable reconstruction of the recorded body landmarks during both terrestrial and aquatic locomotion (see *Data analysis* above), we compared the results of the analysis of inter-segmental coordination obtained from the video camera with those obtained with a high-performance 3D motion-capture system. To this end, in a separate set of experiments, we recorded walking in one dog at a natural speed over-ground using simultaneously two different systems: a) the same Fujifilm camera used for the main study, and b) the SIMI Motion system with 3 cameras (Unterschleissheim, Germany, at 100 Hz). The latter system monitored infrared reflective markers (diameter 2.4 cm) firmly attached to the appropriate anatomical landmarks (Fig 1A). We interpolated the data of SIMI Motion at the same rate as the data acquired with the Fujifilm camera. In a previous study [62], it has been shown that the kinematic data of the sagittal motion of canine hindlimbs during walking obtained with a 2D system correlate well with those obtained with a 3D system, and the data obtained with the 2D system are repeatable. In our analysis, we found that the waveforms of limb segment elevation angles estimated independently by the two recording systems were very similar: the mean RMS difference between the angular waveforms obtained by the two systems was 3±2° (range 1–7°), with the highest difference (7°) for the hand segment and the lowest difference (1°) for the thigh and scapula segments. Nevertheless, the 7° error for the hand segment corresponds to only ∼4.5% error (since the range of motion of the hand segment was ∼150°, see *Results*). Furthermore, an average correlation coefficient between angular waveforms estimated by the two systems was 0.984 (obtained by pooling all segments and all steps together, range 0.985–0.992). These waveforms were also very similar to those obtained during walking in the main experimental session of this dog (when we recorded all three overground gaits using the Fujifilm camera), with an average correlation coefficient of 0.981. Thus, the adequacy of markerless 2D recordings was supported by the low RMS differences and high correlations calculated between the angular waveforms obtained by the two systems and in different experimental sessions. It is also worth noting that, since the main focus of the study was on the inter-segmental coordination estimated by angular motion covariation (principal component analysis, see below), high correlations (∼0.99) resulted in almost identical characteristics of the covariation plane orientation obtained using the two systems.

As a further test of the adequacy of the kinematic analyses employed for dog locomotion, we added a random noise (±1.5 cm) to the time-series of the coordinates of all reconstructed anatomic landmarks in one dog, and verified how this noise affected the parameters of the inter-segmental coordination. We found that the average correlation coefficient between the original and the noisy angular waveforms was 0.99 (all elevation angles and all gaits being pooled together). Moreover, the parameters of the inter-segmental coordination (u_3_ and PV_3_, see below) were very similar: the index of planarity (PV_3_) increased only by ∼0.3% and the azimuth and elevation angles of u_3_ (orientation of the covariation plane) changed only by 4° on average.

### Inter-segmental coordination

The kinematic data were time interpolated over individual gait cycles to fit a normalized 100-point time base. The time course of the elevation angle of each limb segment was expanded into a Fourier series using the fft routine of Matlab. Phase and percent of variance of the first harmonic were computed. The inter-segmental coordination of the elevation angles of hindlimbs and forelimbs segments (thigh, shank, foot, and upper arm, lower arm, hand, respectively) was evaluated in position space as previously described using the principal component analysis [14,35,68]. In humans and primates, the temporal changes of the elevation angles covary during walking [9,14,16,68]. When these angles are plotted in three dimensions (3D), they describe a path that is least-squares fitted to a plane over each gait cycle. Here, we computed the covariance matrix of the ensemble of time-varying elevation angles (after subtraction of their mean value) over each gait cycle. The first two eigenvectors identify the best-fitting plane of angular covariation. The third eigenvector (*u*_3_) is normal to the plane, and defines its orientation. The planarity of the trajectories was quantified by the percentage of total variation (PV_3_) accounted for by the third eigenvector (for ideal planarity, PV_3_ = 0% and the 3^rd^ eigenvalue = 0). For each dog and gait, the parameters of planar covariation (*u*_3_ and PV_3_) were averaged across strides. The *u*_3_ vector was averaged across animals using spherical statistics on directional data, the direction cosines of the mean vector ($\bar{x}$, $\bar{y}$, $\bar{z}$) being defined as [69]:

$$\left(\bar{x},\bar{y},\bar{z}\right)=\left(\frac{S_{x}}{R},\frac{S_{y}}{R},\frac{S_{y}}{R}\right)$$ (2)

where $S_{x}=\sum_{i=1}^{n}x_{i}$, $S_{y}=\sum_{i=1}^{n}y_{i}$, $S_{z}=\sum_{i=1}^{n}z_{i}$, *n* the number of dogs and $R=\sqrt{S_{x}^{2}+S_{y}^{2}+S_{z}^{2}}$.

The inter-subject variability in *u*_3_ and PV_3_ was also assessed. Variability in PV_3_ was assessed as SD across animals. To assess variability of *u*_3_, we calculated the confidence cones centered on the *u*_3_ mean direction. Briefly, for each gait we calculated the points corresponding to the projection of the normal to the plane for each dog onto the unit sphere, the axes of which are the direction cosines with the semi-axis of the thigh, shank, and foot. The 2D distribution of these points for each gait in the plane orthogonal to the *u*_3_ mean direction was quantified using the appropriate scaling factor for the 95% confidence ellipse depending on whether the data had a Fisher or Kent distribution [69,70]. The resulting 95% confidence cones, based on these confidence ellipses [70], were drawn in the 3D space defined by the elevation angles to characterize spatial distribution of the normal to the covariation plane in each gait and for each limb. We also computed the area of these ellipses to quantify the variability of *u*_3_.

### Basic activation patterns of EMG profiles during walk, trot and gallop

To characterize a gait-specific timing of muscle activation and its potential link to the differences in the planar covariation of FL and HL segment motion, we computed the basic activation patterns derived from averaged EMG profiles of 23 extrinsic FL and HL muscles using previously published data from 12 mixed-breed dogs while they walked, trotted and galloped on a level treadmill [59]. Published graphs were scanned, digitized manually taking the touchdown of each limb as time reference, time-interpolated to fit a normalized 100-points time base, and low-pass filtered using a sliding window of ±7 points (in order to avoid the boundary effects, the signal waveform was replicated and concatenated prior to averaging). These data included average EMG recordings from the following ipsilateral muscles: HL (12 muscles)—m. tensor fasciae latae, m. semitendinosus, m. semimembranosus, m. sartorius cranial, m. sartorius caudal, m. rectus femoris, m. gracilis, m. gluteus superficialis, m. gluteus medius, cranial and caudal parts of m. biceps femoris, m. adductor magnus; FL (11 muscles)—m. trapezius p. thoracica, m. trapezius p. cervicalis, m. serratus ventralis thoracis, m. serratus ventralis cervicis, m. rhomboideus, m. pectoralis superficialis transversus, m. pectoralis profundus, m. pectoralis superficialis descendens, m. omotransversarius, m. latissimus dorsi, m. cleidobrachialis.

The hypothesis that *muscle activity profiles* (*m*_*i*_) during different gaits are generated by the nervous system through a linear combination of a small number of basic activation patterns (*p*_*j*_) [54,56] is quantitatively formulated in terms of the equation:

$$m_{i}=\sum_{j}p_{j}\left(t\right)\cdot w_{ij}+residual,$$ (3)

where *w*_*ij*_ are weighting coefficients. We applied a non-negative matrix factorization of the data using the algorithm described by Lee and Seung [71] that constrains the basic patterns and weights to be nonnegative (the rationale was that our data consisted of the non-negative values of rectified EMG activity). To determine the number of significant basic patterns *p*_*j*_, we performed an iterative reconstruction of the EMG profiles using *j* = 1,…,6 patterns, until the cumulative variance accounted for by these patterns was closest to 90%, that is, the residual error accounted for ~10% of data variance.

### Statistics

Descriptive statistics included the calculation of the mean and SD. For each subject, the parameters were averaged across cycles for the subsequent statistical analysis. Shapiro-Wilk test was used to verify the normality distribution of data. One-way (effect of gait) or two-ways (effects of gait and limb) repeated measures ANOVA were used to evaluate differences in general gait parameters and kinematics. Since for galloping we analyzed separately the gaits in which the limb (ipsilateral to the video camera) was acting as the trailing limb or the leading limb, we recorded the trailing limb in 5 dogs and the leading limb in 4 dogs. Missing data for the ANOVA were replaced by the unweighted mean value estimated from all other dogs. If ANOVA resulted in a significant effect for gait (and/or limb when assessed), then post-hoc tests and multiple comparisons analysis were performed by means of Tukey HSD test. In the Results, we report only selected important differences related to general gait parameters and kinematics. One-tailed t-test was used to verify whether the phase lag between the contralateral limbs significantly differed from 50% (symmetrical gaits). Statistics on correlation coefficients was performed on the normally distributed, Z-transformed values. A Matlab Toolbox for circular statistics [72] was used to characterize phases of the first Fourier harmonics of angular waveform and provided a parametric Watson-Williams test to compare them. Statistical analysis of spherical data [69] was used to characterize the mean orientation of the normal to the co-variation plane (see above) and its variability across steps. Reported results are considered statistically significant for p<0.05.

## Results

### General gait parameters

Gait speeds and cycle durations are plotted in Fig 1C and 1D. Swimming was the slowest gait, but its typical speed (1.44±1.15 m/s) was relatively high taking into account water resistance (Fig 1C), and the cycle duration was not significantly different from that of walking (Fig 1D, p = 0.45, Tukey HSD test). A cycle can be divided in two phases: backward (relative to the body) limb endpoint excursion, and forward excursion. During swimming, these two phases can be labeled as a virtual ‘stance’ and ‘swing’ phase respectively, and rearward leg movements are considered as power strokes and forward movements as return movements. During walking, the relative duration of rearward limb movement was longer than 50% of gait cycle, while for all the other gaits it was less than 50% (Fig 1E). There was also asymmetry in this parameter between FL and HL for swimming (symmetrical gait) and for the trailing limb in galloping (p<0.00002 and p = 0.05, respectively, Fig 1E).

**Fig 2:**
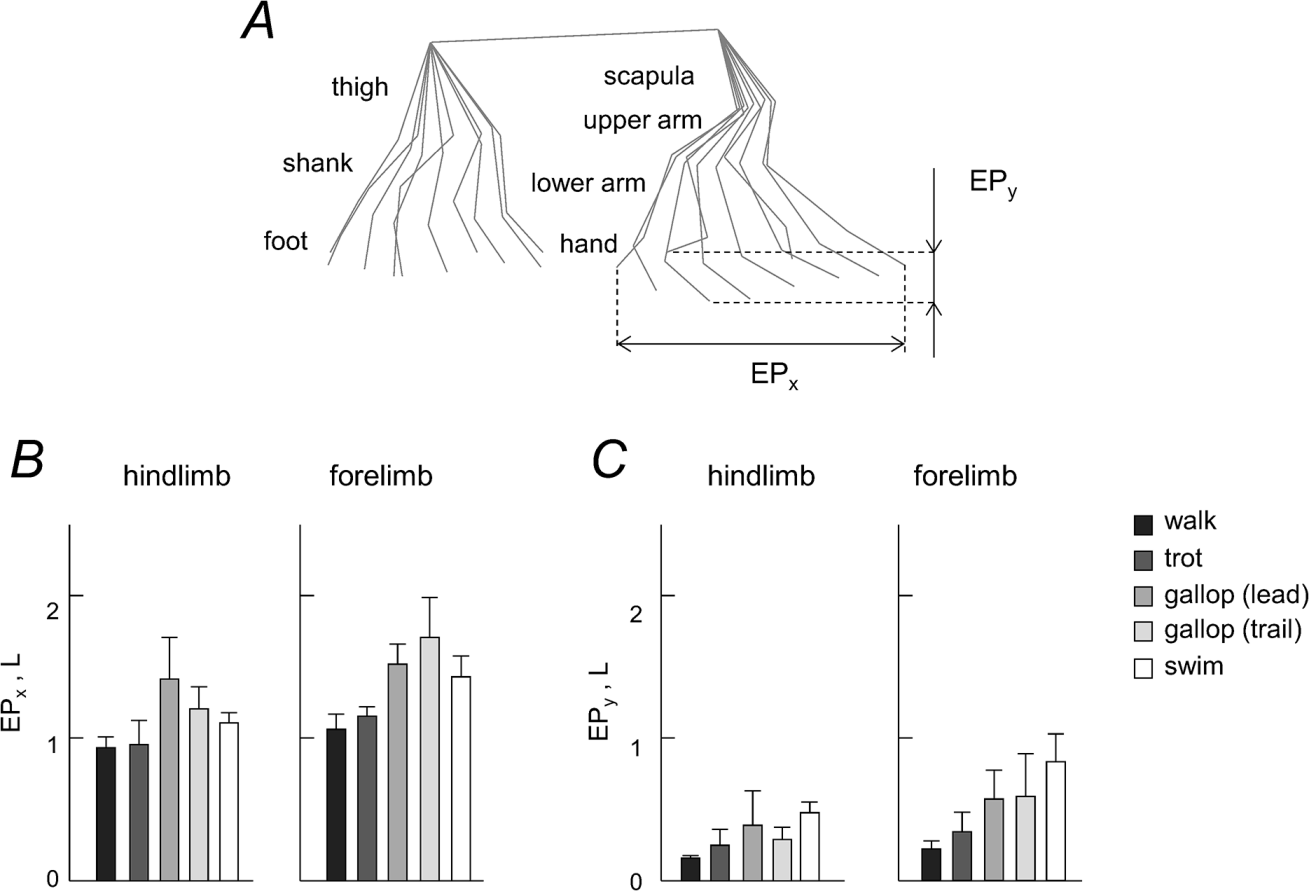
Forelimb and hindlimb endpoint (EP) path. A: stick diagram of a single stride (limb segment movements relative to hip and scapula). EP_x_ and EP_y_ denote horizontal and vertical endpoint excursions, respectively. B and C: mean values (+SD, n = 6) of EP_x_ and EP_y_ excursions for the hindlimb and forelimb.

Fig 2 illustrates horizontal and vertical limb endpoint excursions. Horizontal limb excursions were significantly higher for FL than for HL in all gaits (p<0.03, Tukey HSD tests) except for comparing the leading FL and HL in galloping (p = 0.11) (Fig 2B). Vertical limb excursions were also significantly higher for FL than for HL in all gaits (p<0.04). Moreover, it is worth noting that they exceeded 1 L for the forelimb in swimming, likely in relation to the need to provide a sufficient vertical stroke to avoid sinking and to keep the head outside the water.

### Inter-limb coupling

**Fig 3:**
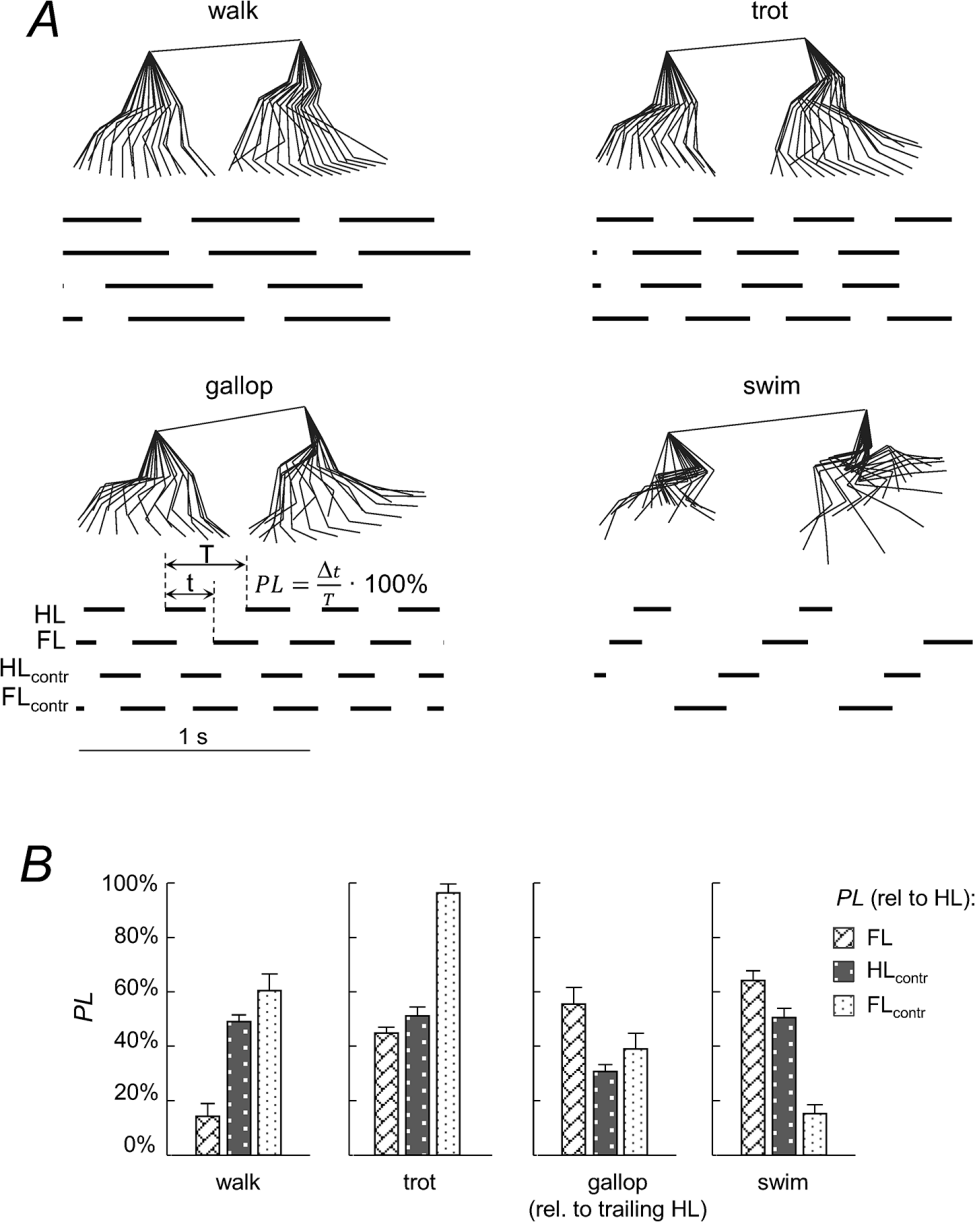
Intra-limb coordination. A: stick diagrams of a single cycle and limb contact patterns of one dog in walk, trot, gallop and swim. Black bars indicate the time of backward limb excursion of each leg: hindlimb on the recording camera side (HL), ipsilateral forelimb (FL), contralateral hindlimb (HL_contr) and contralateral forelimb (FL_contr). *PL*–phase lag between limbs determined as the relative timing of the limb cycle onset (relative to HL) expressed as a percentage of the gait cycle. B: mean *PL* values (+SD, n = 6) for all limbs and gaits.

The fore-aft movement of the limb endpoints was used to determine the gait patterns (Fig 3A and 3B). In general, the dogs maintained a 1:1 frequency relationship between the limbs, despite some inter-stride variability in the onset of footfalls. Fig 3B shows the phase lag (*PL*) between the limbs determined as the relative timing of the limb cycle onset (relative to HL ipsilateral to the recording camera), expressed as a percentage of the gait cycle. For the pairs of contralateral limbs, *PLs* were on average 49.8±1.9% for FLs and 50.2±1.3% for HLs for walking, trotting and swimming, as one would expect for symmetrical gaits, whereas they were significantly smaller than 50% for galloping (18.3±4.5% for FLs and 26±23.1% for HLs, p<0.001 for both limbs, one-tailed t-tests). For the overall pattern during symmetrical gaits, the results showed significant variations in the inter-limb coordination: the canine gait pattern ranged from a lateral sequence of footfalls during walking to a diagonal sequence in trotting and swimming (Fig 3B).

### Joint and segment angular motion

**Fig 4:**
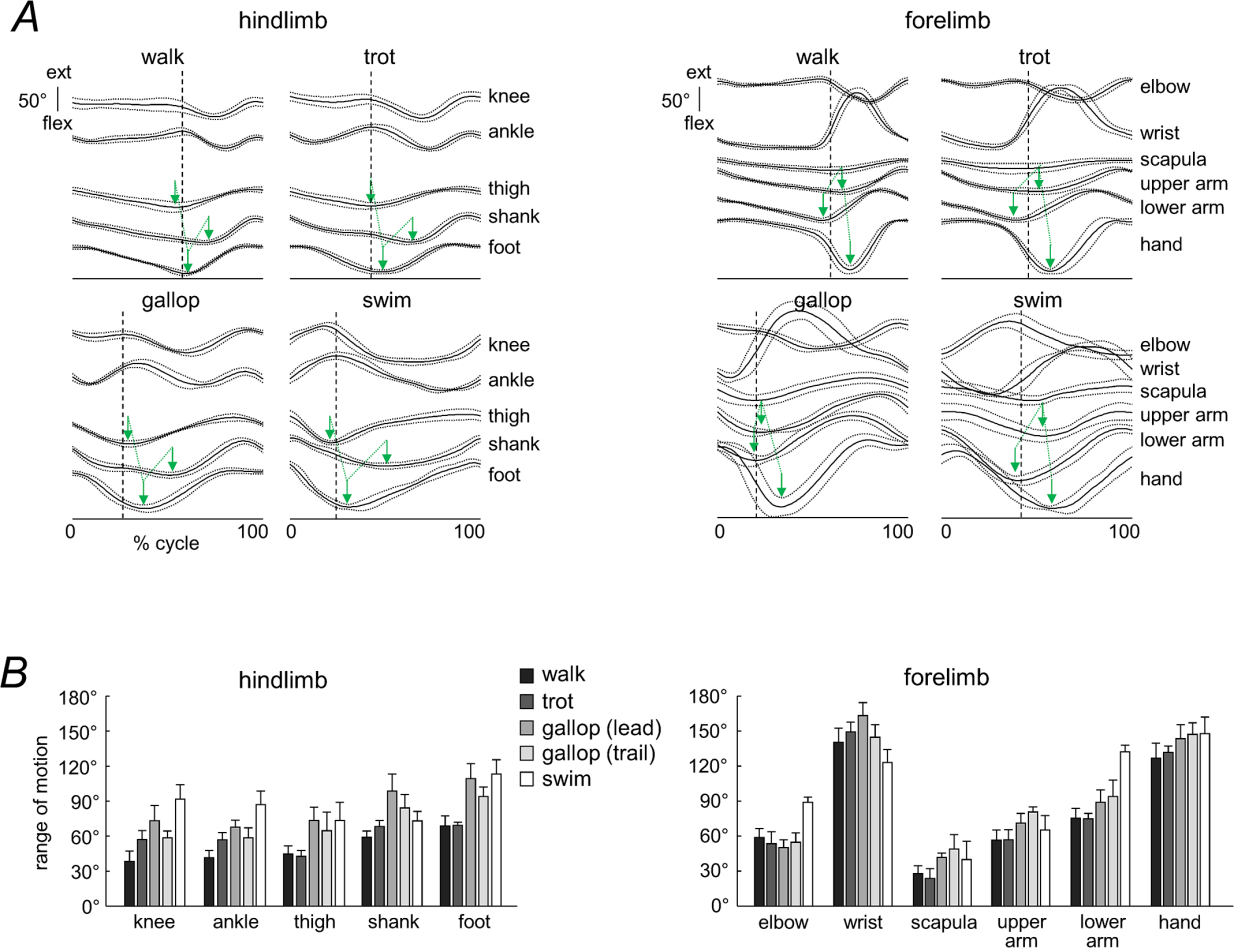
Kinematic patterns. A: ensemble-averaged waveforms (±SD, n = 6) of the limb joint (knee, ankle, elbow, wrist) and segment elevation (thigh, shank, foot, scapula, upper arm, lower arm, hand) angles in the sagittal plane (for galloping, the waveforms for the leading HL are shown). Data are plotted versus normalized gait cycle. Note a similar temporal sequence of minima of the elevation angles across gaits for each limb (schematically marked by green arrows). The dashed vertical lines in each subplot represents the onset of forward limb movement. B: range of angular motion (mean+SD) for HL and FL in different gaits.

Fig 4A shows the average joint and elevation angle profiles (across all dogs) plotted as a function of the normalized gait cycle. In general, the angular waveforms differed across gaits, in particular due to differences in the relative duration of rearward and forward limb movements. In addition, there were differences in the amplitude of angular motion between HL and FL. For instance, the range of motion of the most distal joints differed by as much as ∼2–3 times (ankle vs. wrist), depending on the gait (Fig 4B). For the elevation angles, there was a similar tendency for the most distal segments (foot vs. hand). The smallest range of motion among different elevation angles was observed for the scapula (Fig 4B).

There were also similar features in the kinematic patterns across gaits (Fig 4A). These general features of angular waveforms were also similar to those of walk and trot obtained in the previous studies [30,32,73,74]. The angular waveforms of trot, gallop and swim correlated much better with those of walking if they were time-normalized (stretched) to the same relative stance duration as that of walking (see Methods, Table 2). After normalization, stance duration of the gaits different from walking increased, while the swing duration decreased (according to Fig 1E) to match the same (58% gait cycle) relative stance duration. On average (all segments and gaits being pooled together), the correlation coefficient was 0.54 for non-normalized waveforms and 0.91 for normalized ones. Also, there was a similar temporal sequence of minima in the elevation angles across gaits for each limb (marked schematically by green arrows in Fig 4A).

**Fig 5:**
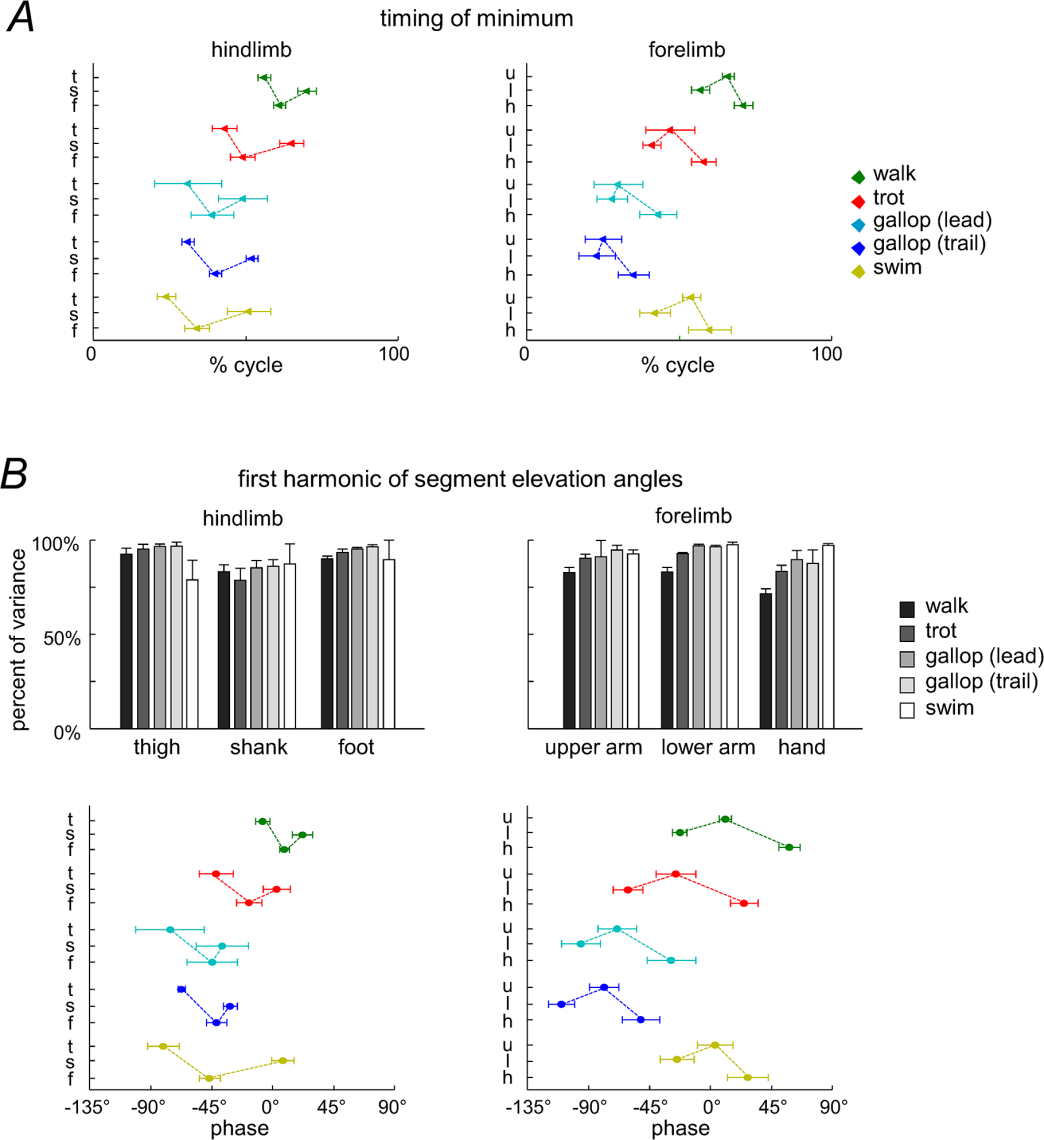
Temporal characteristics of the HL and FL elevation angles. A: timing of minima (triangle) of the elevation angles (±SD). Y axis: t—thigh, s—shank, f–foot, u–upper arm, l–lower arm, h–hand. B: Characteristics of the first harmonic of HL and FL segment elevation angles. Upper panels—percent of variance accounted for by the first harmonic. Lower panels—phase of the first harmonics (circle) (zero refers to the cosine function). Note the phase-lead of the thigh segment in HL and of the lower arm segment in FL in all gaits (consistent with the temporal sequence of minima in the elevation angles, panel A).

On the other hand, the waveforms were substantially different for HL and FL in all gaits (Fig 4A). Fig 5 illustrates the characteristics of the first harmonic and the temporal sequence of the minima in the elevation angles. The first harmonic accounted for a considerable proportion of data variance for all segments and gaits (on average, 87.8%, Fig 5A). Thus, its relative phase captures basic phase relationships of the inter-segmental coordination. Fig 5B (bottom panels) shows that there was a phase-lead of the thigh segment with respect to the other hindlimb segments in all gaits (on average, the phase was 37°±13° for thigh, 57°±9° for shank, and 45°±11° for foot), and a phase-lead of the lower arm segment with respect to the other forelimb segments of FL (on average, 44°±17° for upper arm, 38°±13° for lower arm, and 53°±14° for hand). The parametric Watson-Williams test confirmed that the phases of paired segments—foot vs thigh, shank vs foot, upper arm vs lower arm and hand vs upper arm—were all different (p<0.02) except for foot vs shank of the leading HL in galloping (p = 0.46). Thus, the temporal sequence of the phase of the first harmonic was maintained in all gaits, namely: ‘thigh-foot-shank’ for HL and ‘lower arm-upper arm-hand’ for FL, consistent with a temporal sequence of minima in the elevation angles (Figs 4A and 5B).

**Table 2:** Correlations between angular waveforms during trot, gallop and swim with those during walk.

		non-normalized waveforms			normalized to the same stance duration		
		trot	gallop	swim	trot	gallop	swim
HL	thigh	0.85	0.50	0.31	0.96	0.97	0.95
	shank	0.92	0.53	0.89	0.96	0.94	0.70
	foot	0.85	0.57	0.52	0.98	0.96	0.95
FL	scapula	0.70	-0.26	0.72	0.90	0.65	0.87
	upper limb	0.83	0.14	0.88	0.96	0.84	0.84
	lower arm	0.74	0.12	0.88	0.98	0.93	0.81
	hand	0.70	-0.28	0.73	0.98	0.94	0.91

Normalized angular waveforms for the three gaits (right three columns) were obtained by stretching and interpolating the original waveforms to fit the same relative stance duration as in walking (58% gait cycle).

### Planar covariation of limb segment elevation angles

**Fig 6:**
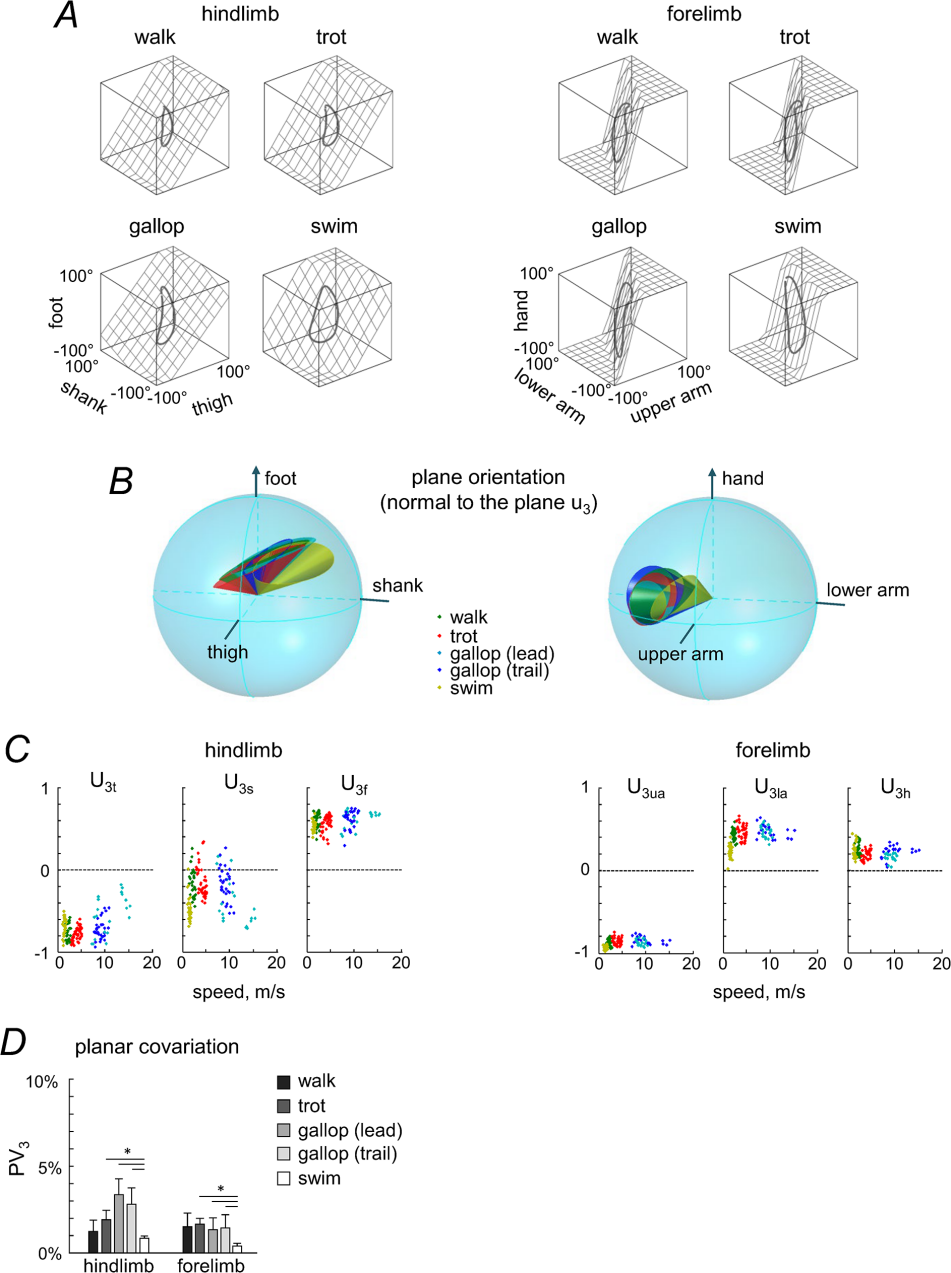
Planar covariation of segment elevation angles in different gaits. A: covariation of limb segment elevation angles during walking, trotting, galloping (trailing limb) and swimming computed for ensemble-averaged elevation angles. The best-fitting plane is shown by grids. B: 95% confidence cones characterizing spatial distribution of the normal to the covariation plane between dogs in each gait and for each limb (left panel–HL, right panel–FL) in the 3D space defined by the elevation angles (see Methods). The foot (and hand for FL) semiaxis is positive, and the shank and thigh (lower and upper arm for FL) semiaxes are negative. Note overlapping of confidence cones in different gaits. C: direction cosines (*u*_3*t*_, *u*_3*s*_, *u*_3*s*_ for HL and *u*_3*ua*_, *u*_3*la*_, *u*_3*h*_ for FL) of the normal to the covariation plane (u_3_ vector) for all individual strides and all dogs versus locomotion speed. D: percent of variance (+SD) accounted for by u_3_ (PV_3_). Asterisks denote significant differences.

Fig 6A shows a three-dimensional view of the ensemble-averaged elevation angles during different gaits. The trajectories progress in the counterclockwise direction; the contact of the foot for HL and hand for FL corresponds to the top of the loops. Planarity was quantified by computing the percentage of variance accounted for by the third eigenvector (PV_3_) of the data covariance matrix: the closer PV_3_ is to 0, the smaller the deviation from planarity. The results demonstrated that the planar covariation was generally maintained for all gaits (PV_3_ = 0.66–3.49%, Fig 6D), though PV_3_ depended on gait (F(4, 20) = 10.204, p = .0001, RM ANOVA). A post-hoc Tukey test showed that PV_3_ was significantly lower in swimming compared to trotting and leading and trailing limbs in galloping (p = 0.007, p = 0.0001 and p = 0.001, respectively, Fig 6D, and tended also to be lower than walking though not-significantly, p = 0.1), consistent with better planarity of the 3D gait loop in the absence of foot-support interactions in humans [75].

The best-fitting planes of the corresponding loop trajectories are illustrated in Fig 6A. The third eigenvector (u_3_) of the covariance matrix is the normal to the plane, and thus characterizes its orientation. Fig 6C shows the direction cosines of the plane normal for all individual strides and dogs as a function of locomotion speed, while Fig 6E presents the direction cosines of the plane normal averaged across dogs for each gait and limb. Despite some inter-stride and inter-subject variability (Fig 6C) and differences in the waveforms of angular motion (Fig 4), the direction cosines of the covariation plane tended to be similar across gaits (Fig 6E). To describe the plane orientation, we also analyzed the 95% confidence cones that characterize the spatial distribution of the normal to the covariation plane between dogs (see Methods). The area of the confidence ellipse represents the inter-subject variability (for HL 0.056, 0.080, 0.095, 0.063 and 0.274 and for FL 0.252, 0.199, 0.157, 0.277 and 0.113 for walk, trot, leading and trailing limbs in gallop and swim, respectively,). There was an overlap of confidence cones across different gaits for each limb (Fig 6B, left panel–HL, right panel–FL), consistent with similarities of the normalized angular waveforms (Table 2, right columns).

**Fig 7:**
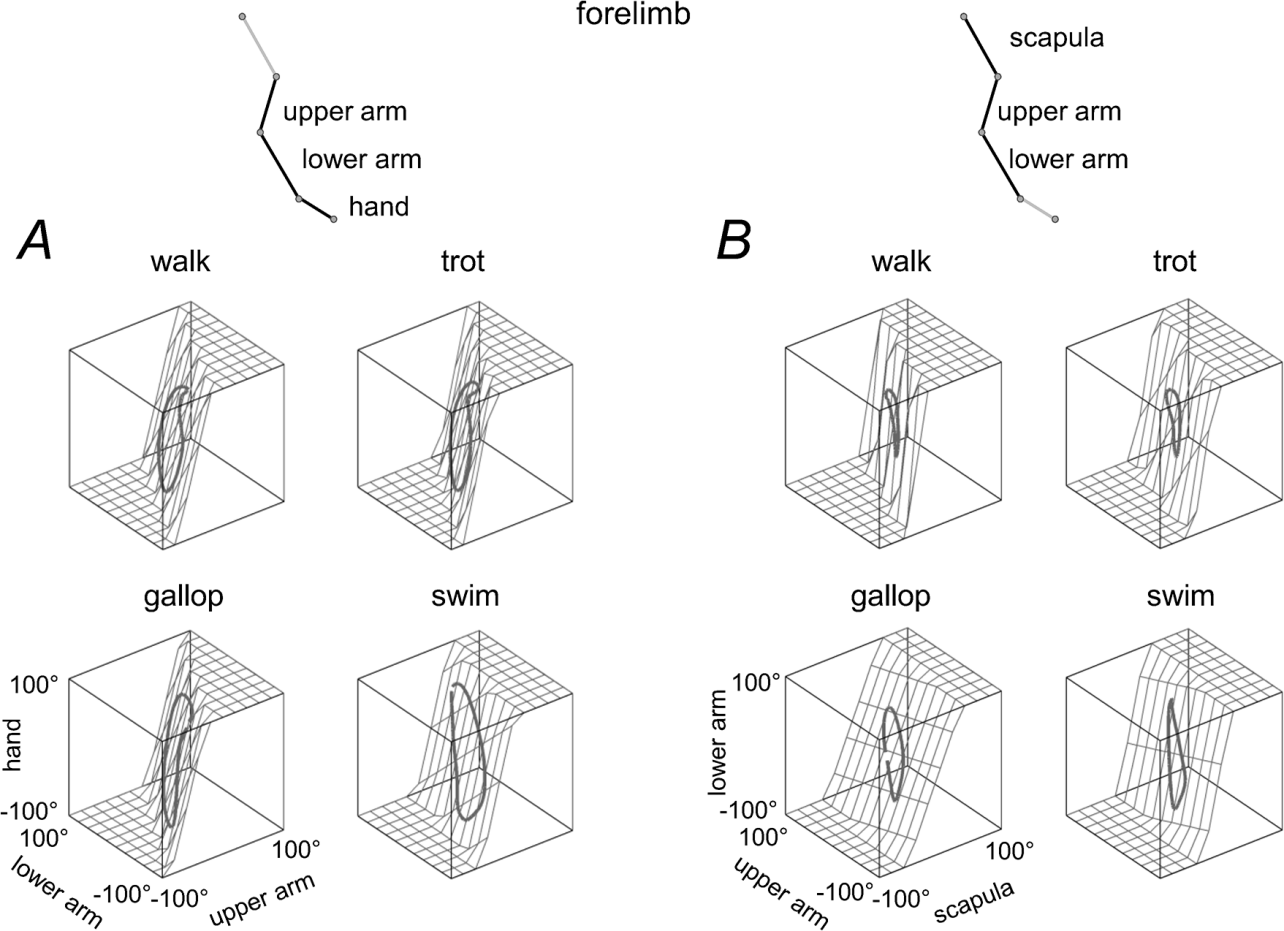
Planar covariation of forelimb segment elevation angles using different tri-segmental FL models. A: *'upper arm—lower arm–hand’* model. B: *‘scapula—upper arm—lower arm’* model. The same format as in the right panel of Fig 6A. Note roughly similar orientation of the covariation plane between these two models of FL (A and B) and different orientation compared to the HL model (Fig 6A).

While the covariation plane orientation tended to be similar for different gaits, it differed systematically between HL and FL (Fig 6B, 6C and 6E). Indeed, the confidence cones of the plane normal did not overlap for HL and FL (Fig 6B), and the mean u_3_ vectors were different: for HL, the elevation angle of u_3_ was 35°, 33°, 25°, 27° and 29°, and the azimuth angle was -180°, -174°, -172°, -161° and -160° in walk, trot, leading and trailing limbs in gallop and swim, respectively, for FL, the elevation angle of u_3_ was 13°, 6°, 6°, 11° and 15°, and the azimuth angle was 156°, 149°, 149°, 147° and 168°, respectively. The analysis of the inter-segmental coordination for the forelimb (Fig 6) was performed using the forelimb segments (upper arm, lower arm and hand) serially homologous to those of the hindlimbs. Since the scapula also undergoes appreciable rotations in the sagittal plane (Fig 4B), in a complementary analysis we verified whether the observed difference in plane orientation between FL and HL (Fig 6A) holds also in an alternative serial tri-segmental forelimb model [67]: scapula—upper arm—lower arm (Fig 7, even though during swimming the reconstruction of scapula is less precise due to vicinity to the water surface). The covariation plane orientation of this alternative FL model (Fig 7B) was more similar to that of the previous FL model than to HL (cf. Figs 6A and 7).

### Decomposition of published EMG data during canine locomotion into basic temporal patterns

**Fig 8:**
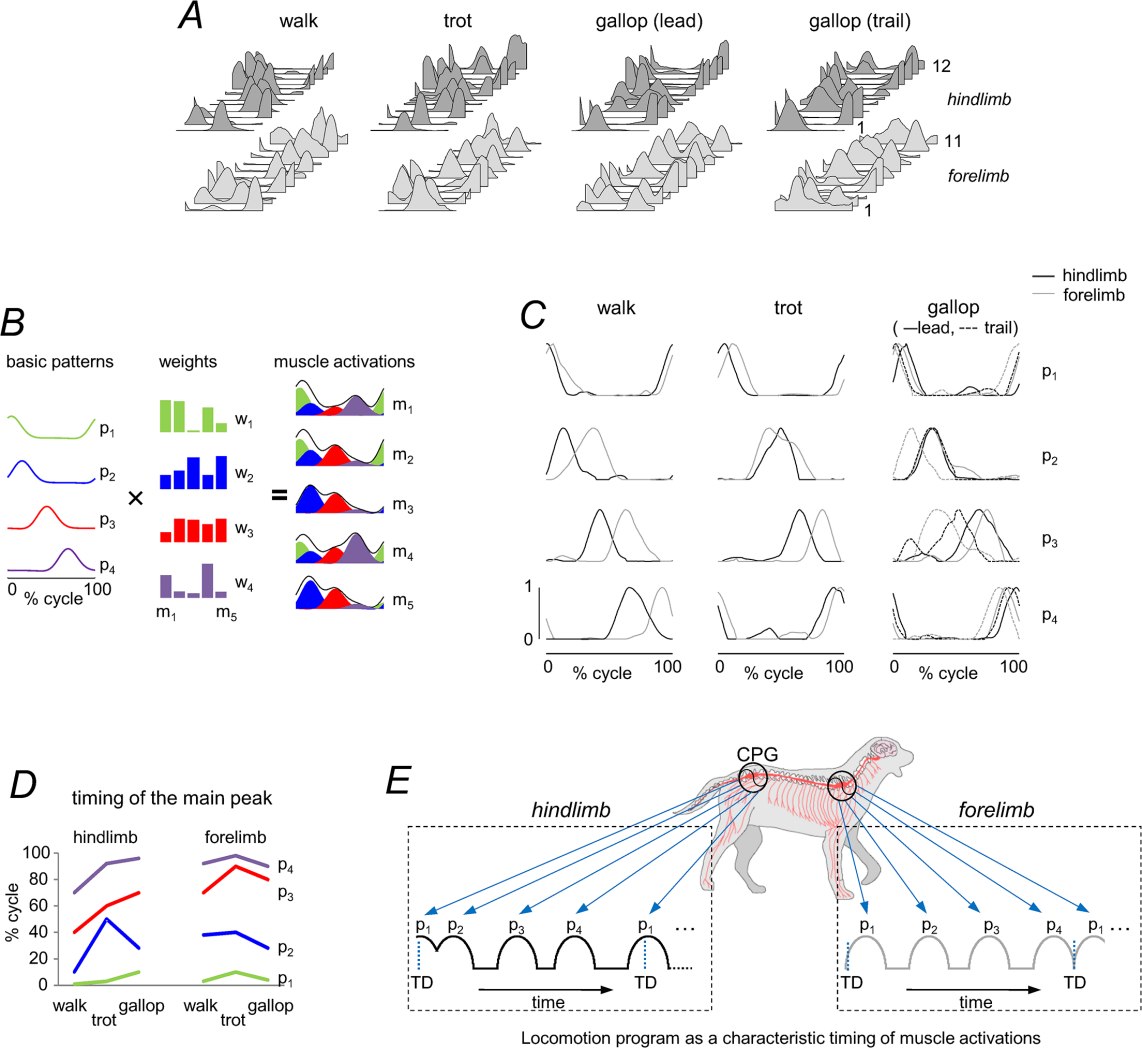
Basic patterns of ipsilateral EMG profiles of 23 extrinsic FL and HL muscles during canine locomotion. A: stride-normalized (with respect to touchdown of each limb), averaged and low-pass EMG data during walk, trot and gallop of twelve mixed-breed dogs taken from [59]. In dark gray—HL, from bottom to top (1–12): m. tensor fasciae latae, m. semitendinosus, m. semimembranosus, m. sartorius cranial, m. sartorius caudal, m. rectus femoris, m. gracilis, m. gluteus superficialis, m. gluteus medius, m. biceps femoris cranial, m. biceps femoris caudal, m. adductor magnus. In light gray–FL, from bottom to top (1–11): m. trapezius p. thoracica, m. trapezius p. cervicalis, m. serratus ventralis thoracis, m. serratus ventralis cervicis, m. rhomboideus, m. pectoralis superfic. transversus, m. pectoralis profundus, m. pectoralis superf. descendens, m. omotransversarius, m. latissimus dorsi, m. clediobrachialis. B: schematic of motor modules. Simulated example of muscle activity profiles as weighted sum of basic patterns (*p*_*j*_): *m*_*i*_ = ∑_*j*_ *p*_*j*_(*t*)⋅*w*_*ij*_ + *residual*. The outputs of the first (green), second (blue), third (red) and forth (violet) modules are summed together to generate overall muscle activation (black envelope). C: basic activation patterns of HL (black) and FL (gray) muscles during walk, trot and gallop. EMG data were decomposed into basic activation patterns using non-negative matrix factorization. For gallop, the patterns for the leading (solid lines) and trailing (dotted lines) limbs were superimposed. The four basic patterns account for ∼90% of variance in each limb and gait and are each characterized by a relatively narrow peak of activation (Gaussian-like) at a particular phase of the cycle (with respect to touchdown of each limb). Components are designated in chronological order. D: the timing of the main peaks of 4 basic patterns across gaits and limbs. Zero timing for HL and FL refers to touchdown of HL and FL, respectively. E: locomotion motor program as a sequence of activation pulses [52,54]. The schematic diagram of activation pulse timings corresponds to those of walk. TD = touchdown. Note different phases of basic muscle activity in HL and FL for each gait.

We examined the patterns of muscle activation from a large set of muscle recordings published by [59], in which the average EMG activity from 12 HL and 11 FL muscles was determined for a standard step cycle during walk, trot and gallop (with respect to touchdown of each limb). Muscle activity tends to occur in bursts with specific timings during a gait cycle (Fig 8A), consistent with ‘drive pulse’ rhythmic elements or primitives in the spinal circuitry of animals [76,77]. Although the activation patterns in the present data set appear muscle specific, there are clearly preferred phases of activation during the gait cycle. To characterize a gait-specific timing of muscle activation, EMG data were decomposed into basic activation patterns (Fig 8B) using non-negative matrix factorization (see Methods). We were especially interested in looking for a temporal correspondence of hypothetical pulsatile burst generators for HL and FL for each gait.

The results showed that four basic temporal patterns accounted for ∼90% of the total waveform variance across the recorded HL and FL muscles for all gaits (range 88–95%). These basic patterns are illustrated in Fig 8C, designated in chronological order of the main peak relative to the gait cycle. For gallop, the patterns for leading and trailing limbs were superimposed, since pairing limbs in the asymmetrical gait is uncertain. Overall, the timing of basic patterns differed between walk, trot and gallop, as it happens for human walking and running in relation with corresponding changes in the relative stance/swing duration [54]. However, it is worth stressing that, for each gait, there are different phases of muscle activity with respect to touch-down of HL and FL (Fig 8C), in accordance with the previously described differences in the planar covariation of limb segment motion (Fig 6). Fig 8D summarizes a schematic representation of the canine motor program for walking as a sequence of activation pulses [52,55,76].

## Discussion

We examined the kinematics of HL and FL movements during quadrupedal locomotion in dogs. A novel finding was that all limbs exhibited the planar constraint of the inter-segmental coordination, even though FL and HL substantially differ in musculoskeletal anatomy and show opposing phase relationships and limb-specific covariation plane orientation (Figs 4–6). The analysis of published muscle activity patterns also suggested a characteristic limb-specific phase control of muscle activation for each gait (Fig 8). Below we discuss the findings in the context of limb-specific control of the inter-segmental coordination in different gaits.

### Kinematics of terrestrial and aquatic locomotion

The rationale of our study was to compare various forms of quadrupedal locomotion. Swimming is a relatively frequent behavior among mammals, normally performed when they actively cross short stretches of water. It is valuable to place the locomotion in water in context with that of terrestrial creatures, swimmers, and fliers, and so contribute to the emerging integrative view of biolocomotion [4,78–80]. For instance, locomotion in water in some terrestrial insects, which occasionally swim, shows a stereotyped tripod pattern similar to that used by many insects for running on land [81]. Estimations of the forward thrust from 3D recordings of insect leg movements showed that thrust was mainly produced by the front legs (and to a lesser extent by the middle legs), while the hind legs contributed drag [81]. Nevertheless, several arthropods exhibit different modes of locomotion in/on water and on land. Typically, during the power stroke, the front limbs of swimming animals are extended and moved rapidly, whereas in the return stroke they move slower and are held closer to the body [4]. The hind legs contribute only little to the overall thrust, suggesting that they mainly serve for stabilization and steering. The data on swimming mammals are still somewhat limited [82,83].

Aquatic locomotion represents an interesting example of body movements without the ground constraint due to a common support for the limbs during stance. Our results showed that the sequence of footfalls was basically reversed in walking and swimming: HL-FL-HL_contr_-FL_contr_ in walking and FL-HL-FL_contr_-HL_contr_ in swimming (Fig 3). Overall, the canine gait pattern was characterized by a lateral sequence of footfalls during walking and a diagonal sequence in swimming (Fig 3). Early tetrapods from the Devonian period exhibited diagonal-sequence locomotion [84], suggesting that this type of quadrupedalism is a very old locomotor trait [85], has been preserved for millions of years and may involve both biomechanical and behavioral advantages [86].

For swimming, we observed a high planarity of inter-segmental coordination (PV_3_ < 1%, Fig 6). The limb strokes for swimming had significantly larger amplitudes than those of walking and trotting (Fig 2). Some variations in the covariation plane orientation across gaits or steps (Fig 6) could be explained in part by the effect of speed, also observed in humans [68]. Nevertheless, it is remarkable that, despite substantial changes in the duration and amplitude of angular motion (Fig 4) and variable inter-limb phase patterns (Fig 3), the intra-limb coordination pattern was fairly well conserved in different gaits (Fig 6). This apparent discrepancy may reflect different degrees of flexibility of phase connections of neural oscillators that control inter-limb and inter-segment coordination in different gaits. Similarities in the inter-segmental phase pattern across gaits may also explain a relatively rapid dog’s adaptation to aquatic locomotion (as soon as a dog is plunged into water it can swim), since a similar locomotor program may underlie phase relationships. In contrast, albeit arm and legs are coordinated [87], humans display quite different styles of coordination during swimming and they typically need to learn how to do it.

### Inter-segmental coordination in forelimbs and hindlimbs

Our results extend to canine locomotion previous observations in humans, monkeys and cats that the temporal changes of limb segments with respect to the direction of gravity co-vary according to a law of planar co-variation during a variety of tasks [9,16,35,37,88–91].

**Fig 9:**
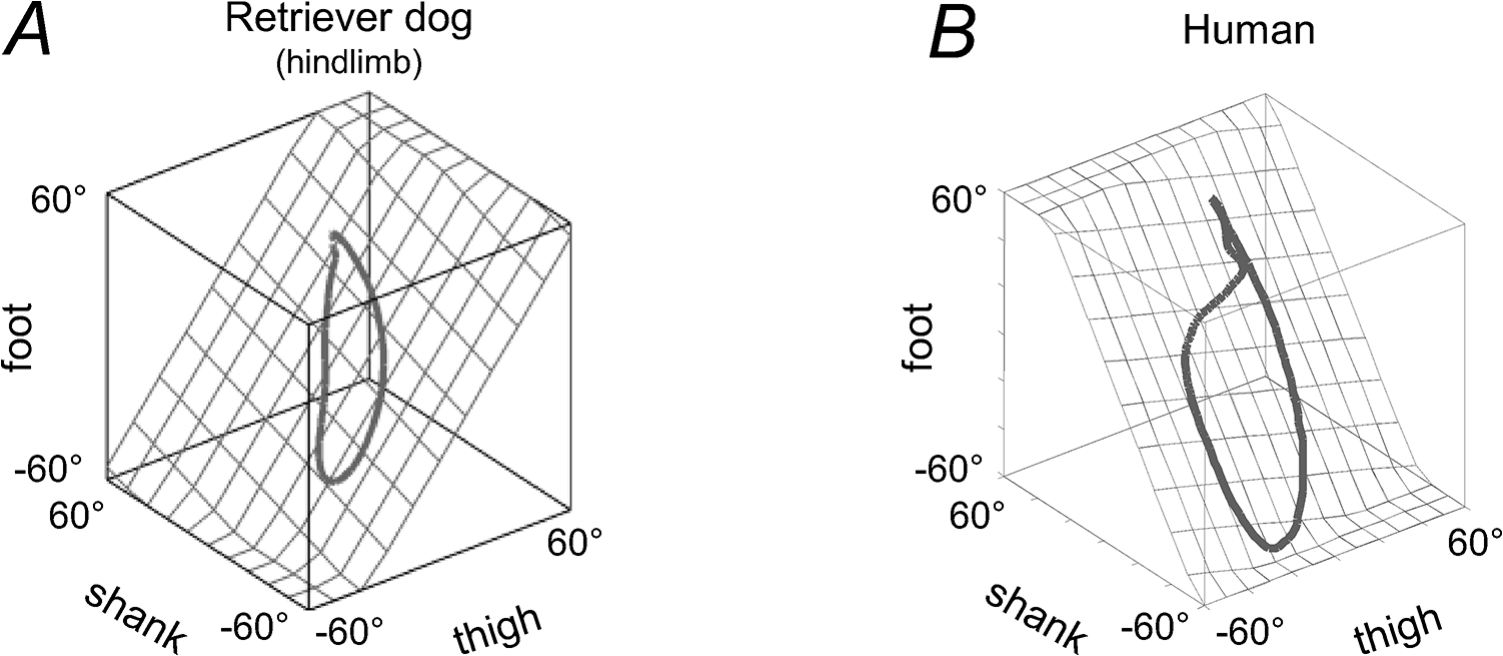
Pattern of inter-segmental coordination in human (A) and canine (B) gait. Covariation of thigh, shank, and foot elevation angles is shown during walking at a natural speed. Gait loops of each species are represented with respect to the best-fitting planes (grids). Note different covariation plane orientation in human and dog locomotion, attributable to different phase relationships between limb segment oscillations. Data from human were adapted from [92].

The spatial orientation of the covariation plane of the hindlimb of dogs is very different from that of human lower limbs (cf. Fig 6 with Fig 2 in [92]. Even though biomechanics contributes to the emergence of the planar covariation of limb segment motion [92], the orientation of the covariation plane reflects basic phase relationships between segment motions, and adaptation to different walking conditions [68,93]. For instance, the orientation of the planar covariation shows systematic changes with walking speed [68] and in different gaits (running, staircase and uphill walking, obstacle avoidance, etc.) in adults [16,89,94,95], while the orientation is relatively fixed in different gaits in toddlers at the beginning of independent walking [93], suggesting its neural control rather than simple biomechanical consequence. Moreover, the planar covariance is markedly different in human newborns [77] and in adults with a spinal cord lesion [92]. In prosthetic gait of transfemoral amputees, the best-fitting plane rotates around the long axis of the gait loop with increasing walking speed even more for the sound limb of expert amputees than in control subjects [94]. The authors of this latter study suggested that their results reveal a centrally commanded compensation strategy. It is also worth stressing that the orientation of the covariation plane is different between dogs and humans (Fig 9), further suggesting different phase relationships between segment movements. Finally, the modelling studies confirmed changes in the covariation plane orientation of simple mechanical oscillators with appropriate phase shifts [17]. In particular, the difference in the orientation between dogs and humans (Fig 9) might be attributable to a digitigrade (dogs) vs. plantigrade (humans) gait [9,16]. In fact, the correlation between the foot and shank elevation angles was much lower in dogs (Fig 4A) than that in humans. The current results therefore suggest that the strategy by which the central nervous system achieves inter-segmental coordination and adapts its spatiotemporal structure in dogs (and non-human primates) differs somewhat from the kinematic principles that operate in human gait control, consistent with early studies on variances in the distal limb segment control in humans and dogs [22].

Interestingly, we observed a limb-specific inter-segmental coordination pattern (Figs 4–7) likely due to a different parametric tuning in the phase-relationship of inter-segmental coordination between FL and HL. Despite differences in inter-limb coupling (the frequent use of diagonal-couplet interlimb timing in primates), the organization of intra-limb coordination during walking in dogs shows a number of similarities with primates [9], suggesting that common principles may operate during stepping among a wide range of mammals. In particular, the orientation of the covariation plane is also different between FL and HL in Rhesus monkey gait (cf. our Fig 6A with Fig 6A in [9]). The general features of angular waveforms (Fig 4A) were also strikingly similar to those obtained in previous studies of dog kinematics during walk and trot [32,73,74], suggesting that they are characteristic for each joint in dogs of similar morphology [73].

The key anatomical inter-limb difference consists in the orientation of the forelimb and hindlimb; the elbow is facing posteriorly and the knee joint anteriorly, and the scapula is either held by the clavicle or, in most mammalian groups, completely freed from any connection with the trunk [67]. This distinctive functional orientation of limb segments may impose specific parametric tuning in the phase-relationships and specific control of FL and HL segments. The theory of coupled oscillators is a useful tool for studying synchronized, periodic dynamics of physical and biological systems [44,96], based on specific phase resetting and the stabilization of an isolated cycle as the attractor of animal dynamics [10,97]. For instance, the fact that a temporal sequence of minima in the elevation angles around the stance-to-swing transition was different for each limb (Figs 4A and 5) may indicate that the so-called ‘leading’ segment [98] is also different for the HL and FL control: proximal segment (thigh) for HL and intermediate segment (lower arm) for FL. Barliya et at. [17] introduced a mathematical model that represented the rotations of the elevation angles in terms of simple oscillators with appropriate phase shifts between them. Again, such analysis showed a clear phase-lead of the first harmonic of the thigh and lower arm segments for HL and FL, respectively (Fig 5B), consistent with different covariation plane orientation (Fig 6) and different ‘leading’ segments [98] for HL and FL.

### Hypothetical limb-specific control of inter-segmental phase patterns

Differences in the intra-limb coordination strategy (Fig 6) must also be taken into consideration when studying the coupling of neural oscillators with limb mechanical oscillators [35,37]. If one accepts that the inter-segmental coordination pattern can be controlled by symmetrically organized unit burst generators for each joint or synergistic sets of muscles [17,42], the observed findings (Figs 4A, 5B, 6A and 6B) may point to the differences in the organization for cervical and lumbosacral central pattern generators. In particular, it has been proposed that a multi-layered organization of mammalian locomotor CPG includes a rhythm-generating layer and a pattern-generating layer [55,99–101], and the locomotor output can be represented as a characteristic timing of muscle activation [52]. Furthermore, the descending cortical signal interacts with the interneuronal networks in the spinal cord to ensure the appropriate limb movement control and the basic locomotor rhythm [102–104]. Subpopulations of motor cortical neurones, active sequentially during the step cycle, may regulate the activity of small groups of synergistic muscles during quadrupedal locomotion, and these synergies, identified by a cluster analysis, are defined by periods of muscle activity that are coextensive [103].

An interesting approach to capture the essence of biological input/output transformations consists in analyzing the spatiotemporal characteristics of the spinal motor output and modelling the locomotor control system using artificial neural networks [45,46,51,55]. The results of this approach demonstrate the ability of neural networks to model the transformation between a kinematic movement plan and the necessary muscle activations [48], even though they do not directly specify the actual structures involved. The dynamic behavior of the musculo-skeletal system can be modelled through a linear combination of a small number of basic muscle activation patterns linked to specific kinematic events [48,51]. Both supraspinal input and sensory information have a major role in determining the timing of the motor activation patterns during steady state locomotion [99,105].

Our analysis of available published data for canine locomotion [59] showed that multi-muscle activity patterns of both HL and FL (Fig 8A) can be decomposed into a small set of 4 basic temporal patterns that account for ~90% of total variance, and are each characterized by a relatively narrow peak of activation at a particular phase of the cycle (Fig 8C and 8D). Their timing is not invariant but differs between different gaits in conjunction with changes in the relative stance/swing duration (Fig 1E), as it does for human walking and running [54]. Since we were mainly interested in the temporal structure of the motor patterns, the decomposition analysis was applied to the normalized EMG profiles (they were scaled to the maximum amplitude observed during each gait [59]). Further investigations are needed to elucidate muscle synergies (weights of basic activation patterns, Fig 8B) common to each gait mode. However, it is worth stressing that, for each gait, there are different phases of muscle activity with respect to touchdown of HL and FL (Fig 8C).

The previous works also revealed some limb-specific features in the organization and coupling between FL and HL controllers [20,106–109]. Both descending and ascending connections between cervical and lumbosacral CPGs, as well as an intrinsic rhythmogenesis capacity of the thoracic spinal network, have been described in quadrupedal animals [110]. Nevertheless, there are essential differences in their neurotransmitter systems [108] and in the inter-limb influences [20]. For instance, forelimb movements in the dog may facilitate or even trigger hindlimb stepping while the opposite influences are much weaker [20]. Inter-limb coordination may also reflect supraspinal control; hindlimb-related neurons in motor cortex respond to changes in forelimb movements during locomotion and vice versa, although the percentage of neurons from the HL and FL area that are modulated by motion of the other pair of limbs is very different [111]. In sum, the observed limb-specific inter-segmental phase pattern (Figs 4–8) may be in accordance with the specific biomechanical function of FL and HL.

Finally, it is worth noting that the intra-limb coordination pattern is relatively conserved (Figs 5 and 6) while muscle activations tend to intervene during different time epochs in different gaits (Fig 8), consistent with the idea that EMG commands are subservient to the kinematic reference. Therefore, the coupling of neural oscillators with limb mechanical oscillators may be more complex than it was previously thought, since the rhythm-generating layer or ‘time-keeping function’ of the CPG for locomotion [51,55,99] appear to be kinematically-driven. Therefore, although it is often assumed that CPGs control patterns of muscle activity, another hypothesis is that they control patterns of limb segment motion instead [17,37].

## Supporting Information

S1 Dataset A compressed archive (.zip) contains two folders and a text file with a detailed description of the data organization. S1_Dataset.zip contains two folders: 1) Files in the 'Dogs Data' folder are the kinematic data in Matlab format (.mat) for each subject and each gait; 2) Files in the 'comparison' folder are the data used for the validation of the markerless motion capture system.
(ZIP)

## References

[1] Hildebrand M. Symmetrical gaits of primates. Am J Phys Anthropol. 1967;26: 119–130. 10.1002/ajpa.1330260203

[2] Cavagna GA, Heglund NC, Taylor CR. Mechanical work in terrestrial locomotion: two basic mechanisms for minimizing energy expenditure. Am J Physiol. 1977;233: R243–261. 411381 10.1152/ajpregu.1977.233.5.R243

[3] Goslow GE, Seeherman HJ, Taylor CR, McCutchin MN, Heglund NC. Electrical activity and relative length changes of dog limb muscles as a function of speed and gait. J Exp Biol. 1981;94: 15–42. 7310312 10.1242/jeb.94.1.15

[4] Alexander R. Locomotion of animals Glasgow: Blackie; 1982.

[5] Blickhan R. The spring-mass model for running and hopping. J Biomech. 1989;22: 1217–1227. 2625422 10.1016/0021-9290(89)90224-8

[6] Sefati S, Neveln ID, Roth E, Mitchell TRT, Snyder JB, MacIver MA, et al Mutually opposing forces during locomotion can eliminate the tradeoff between maneuverability and stability. Proc Natl Acad Sci U S A. 2013;110: 18798–18803. 10.1073/pnas.1309300110 24191034 PMC3839770

[7] Carrier DR, Gregersen CS, Silverton NA. Dynamic gearing in running dogs. J Exp Biol. 1998;201: 3185–3195. 9808832 10.1242/jeb.201.23.3185

[8] Gatesy SM, Dial KP. Locomotor Modules and the Evolution of Avian Flight. Evolution. 1996;50: 331 10.2307/2410804 28568886

[9] Courtine G, Roy RR, Hodgson J, McKay H, Raven J, Zhong H, et al Kinematic and EMG Determinants in Quadrupedal Locomotion of a Non-Human Primate (Rhesus). J Neurophysiol. 2005;93: 3127–3145. 15647397 10.1152/jn.01073.2004

[10] Holmes P, Full RJ, Koditschek D, Guckenheimer J. The Dynamics of Legged Locomotion: Models, Analyses, and Challenges. SIAM Rev. 2006;48: 207–304. 10.1137/S0036144504445133

[11] Riskin DK, Willis DJ, Iriarte-Díaz J, Hedrick TL, Kostandov M, Chen J, et al Quantifying the complexity of bat wing kinematics. J Theor Biol. 2008;254: 604–615. 10.1016/j.jtbi.2008.06.011 18621062

[12] Maes L, Abourachid A. Gait transitions and modular organization of mammal locomotion. J Exp Biol. 2013;216: 2257–2265. 10.1242/jeb.082149 23531814

[13] Mah CD, Hulliger M, Lee RG, O’Callaghan IS. Quantitative analysis of human movement synergies: constructive pattern analysis for gait. J Mot Behav. 1994;26: 83–102. 10.1080/00222895.1994.9941664 15753062

[14] Borghese NA, Bianchi L, Lacquaniti F. Kinematic determinants of human locomotion. J Physiol. 1996;494 (Pt 3): 863–879. 8865081 10.1113/jphysiol.1996.sp021539 PMC1160684

[15] Full R, Kubow T, Garcia M, Schwind W, Koditschek D. Can a simple neural oscillator generate rapid running in cockroaches? Toronto, ON, Canada; 2003.

[16] Ivanenko YP, Cappellini G, Dominici N, Poppele RE, Lacquaniti F. Modular control of limb movements during human locomotion. J Neurosci. 2007;27: 11149–11161. 10.1523/JNEUROSCI.2644-07.2007 17928457 PMC6672838

[17] Barliya A, Omlor L, Giese MA, Flash T. An analytical formulation of the law of intersegmental coordination during human locomotion. Exp Brain Res. 2009;193: 371–385. 10.1007/s00221-008-1633-0 19034442

[18] Sherrington CS. Flexion-reflex of the limb, crossed extension-reflex, and reflex stepping and standing. J Physiol. 1910;40: 28–121. 16993027 10.1113/jphysiol.1910.sp001362 PMC1533734

[19] Arshavsky II, Kots IM, Orlovsky GN, Rodionov IM, Shik ML. Study of biomechanics of running dogs. Biophysics. 1965;10: 665–671. 5868375

[20] Shik ML, Orlovsky GN. Coordination of the legs during a dog’s run. Biophysics. 1965;10: 1037–1047. 5873148

[21] Jayes AS, Alexander RM. Mechanics of locomotion of dogs (Canis familiaris) and sheep (Ovis aries). J Zool. 1978;185 Pt 3: 289–308. 700246 10.1111/j.1469-7998.1978.tb03334.x

[22] Charteris J, Leach D, Taves C. Comparative kinematic analysis of bipedal and quadrupedal locomotion: a cyclographic technique. J Anat. 1979;128: 803–819. 489468 PMC1232882

[23] Afelt Z, Błaszczyk J, Dobrzecka C. Speed control in animal locomotion: transitions between symmetrical and nonsymmetrical gaits in the dog. Acta Neurobiol Exp (Warsz). 1983;43: 235–250. 6660051

[24] Cavagna GA, Franzetti P, Heglund NC, Willems P. The determinants of the step frequency in running, trotting and hopping in man and other vertebrates. J Physiol. 1988;399: 81–92. 3404473 10.1113/jphysiol.1988.sp017069 PMC1191653

[25] Lee DV, Bertram JE, Todhunter RJ. Acceleration and balance in trotting dogs. J Exp Biol. 1999;202: 3565–3573. 10574733 10.1242/jeb.202.24.3565

[26] Bertram JE, Lee DV, Case HN, Todhunter RJ. Comparison of the trotting gaits of Labrador Retrievers and Greyhounds. Am J Vet Res. 2000;61: 832–838. 10895909 10.2460/ajvr.2000.61.832

[27] Walter RM, Carrier DR. Ground forces applied by galloping dogs. J Exp Biol. 2007;210: 208–216. 10.1242/jeb.02645 17210958

[28] Maes LD, Herbin M, Hackert R, Bels VL, Abourachid A. Steady locomotion in dogs: temporal and associated spatial coordination patterns and the effect of speed. J Exp Biol. 2008;211: 138–149. 10.1242/jeb.008243 18083742

[29] Schilling N, Fischbein T, Yang EP, Carrier DR. Function of the extrinsic hindlimb muscles in trotting dogs. J Exp Biol. 2009;212: 1036–1052. 10.1242/jeb.020255 19282501

[30] Fischer MS, Lilje KE. Dogs in Motion. Frankh—Kosmos Verlag Stuttgart; 2011.

[31] Hudson PE, Corr SA, Wilson AM. High speed galloping in the cheetah (Acinonyx jubatus) and the racing greyhound (Canis familiaris): spatio-temporal and kinetic characteristics. J Exp Biol. 2012;215: 2425–2434. 10.1242/jeb.066720 22723482

[32] Orlovsky GN, Shik ML. On standard elements of cyclic motion. Biophysics. 1965;10: 847–854. 5870855

[33] Orlovsky GN, Severin FV, Shik ML. The effect of speed and load on the coordination of movement in running in the dog. Biophysics. 1966;11: 364–366. 6002287

[34] Misiaszek JE, Barclay JK, Brooke JD. Inhibition of canine H reflexes during locomotor-like rotation about the knee arises from muscle mechanoreceptors in quadriceps. J Neurophysiol. 1995;73: 2499–2506. 7666155 10.1152/jn.1995.73.6.2499

[35] Lacquaniti F, Ivanenko YP, Zago M. Kinematic control of walking. Arch Ital Biol. 2002;140: 263–272. 12228979

[36] Shen L, Poppele RE. Kinematic analysis of cat hindlimb stepping. J Neurophysiol. 1995;74: 2266–2280. 8747190 10.1152/jn.1995.74.6.2266

[37] Lacquaniti F, Grasso R, Zago M. Motor Patterns in Walking. News Physiol Sci. 1999;14: 168–174. 11390844 10.1152/physiologyonline.1999.14.4.168

[38] Grillner S. Neuroscience. Human locomotor circuits conform. Science. 2011;334: 912–913. 10.1126/science.1214778 22096178

[39] Kiehn O. Development and functional organization of spinal locomotor circuits. Curr Opin Neurobiol. 2011;21: 100–109. 10.1016/j.conb.2010.09.004 20889331

[40] Guertin PA. Central pattern generator for locomotion: anatomical, physiological, and pathophysiological considerations. Mov Disord. 2013;3: 183 10.3389/fneur.2012.00183 PMC3567435 23403923

[41] Lacquaniti F, Ivanenko YP, d’ Avella A, Zelik KE, Zago M. Evolutionary and developmental modules. Front Comput Neurosci. 2013;7: 61 10.3389/fncom.2013.00061 23730285 PMC3656358

[42] Grillner S. Control of locomotion in bipeds, tetrapods and fish Handbook of Physiology: Section 1: The Nervous System, volume II, Part1 Motor Control. American Physiological Society Bethesda, MD: Brooks Vernon B., Brookhart John M., Mountcastle Vernon B.; 1981 pp. 1179–1236.

[43] Grillner S. Biological pattern generation: the cellular and computational logic of networks in motion. Neuron. 2006;52: 751–766. 10.1016/j.neuron.2006.11.008 17145498

[44] Golubitsky M, Stewart I, Buono PL, Collins JJ. Symmetry in locomotor central pattern generators and animal gaits. Nature. 1999;401: 693–695. 10.1038/44416 10537106

[45] Wootten ME, Kadaba MP, Cochran GVB. Dynamic electromyography. I. Numerical representation using principal component analysis. J Orthop Res. 1990;8: 247–258. 10.1002/jor.1100080214 2303958

[46] Kelly MF, Parker PA, Scott RN. The application of neural networks to myoelectric signal analysis: a preliminary study. IEEE Trans Biomed Eng. 1990;37: 221–230. 10.1109/10.52324 2328997

[47] Srinivasan S, Gander RE, Wood HC. A movement pattern generator model using artificial neural networks. IEEE Trans Biomed Eng. 1992;39: 716–722. 10.1109/10.142646 1516938

[48] Prentice SD, Patla AE. Modelling of Some Aspects of Skilled Locomotor Behaviour Using Artificial Neural Networks In: Computational intelligence for movement sciences: Neural networks, support vector machines, and other emerging technology. Begg R and Palaniswami M (eds). Hershey PA: Idea Group Inc., USA; 2006 pp. 172–196.

[49] Patla AE. Analytic approaches to the study of outputs from central pattern generators In: Neural control of rhythmic movements in vertebrates. Cohen A (ed). New York: John Wiley & Sons; 1988 pp. 455–486.

[50] Olree KS, Vaughan CL. Fundamental patterns of bilateral muscle activity in human locomotion. Biol Cybern. 1995;73: 409–414. 7578478 10.1007/BF00201475

[51] Prentice SD, Patla AE, Stacey DA. Modelling the time-keeping function of the central pattern generator for locomotion using artificial sequential neural network. Med Biol Eng Comput. 1995;33: 317–322. 7475369 10.1007/BF02510506

[52] Ivanenko YP, Poppele RE, Lacquaniti F. Motor control programs and walking. The Neuroscientist. 2006;12: 339–348. 10.1177/1073858406287987 16840710

[53] Neptune RR, Clark DJ, Kautz SA. Modular control of human walking: a simulation study. J Biomech. 2009;42: 1282–1287. 10.1016/j.jbiomech.2009.03.009 19394023 PMC2696580

[54] Cappellini G, Ivanenko YP, Poppele RE, Lacquaniti F. Motor patterns in human walking and running. J Neurophysiol. 2006;95: 3426–3437. 16554517 10.1152/jn.00081.2006

[55] Lacquaniti F, Ivanenko YP, Zago M. Patterned control of human locomotion. J Physiol. 2012;590: 2189–2199. 10.1113/jphysiol.2011.215137 22411012 PMC3424743

[56] Ivanenko YP, Cappellini G, Poppele RE, Lacquaniti F. Spatiotemporal organization of alpha-motoneuron activity in the human spinal cord during different gaits and gait transitions. Eur J Neurosci. 2008;27: 3351–3368. 10.1111/j.1460-9568.2008.06289.x 18598271

[57] Cappellini G, Ivanenko YP, Poppele RE, Lacquaniti F. Motor Patterns in Human Walking and Running. J Neurophysiol. 2006;95: 3426–3437. 10.1152/jn.00081.2006 16554517

[58] Maclellan MJ, Ivanenko YP, Massaad F, Bruijn SM, Duysens J, Lacquaniti F. Muscle activation patterns are bilaterally linked during split-belt treadmill walking in humans. J Neurophysiol. 2014;111: 1541–1552. 10.1152/jn.00437.2013 24478155 PMC4035776

[59] Deban SM, Schilling N, Carrier DR. Activity of extrinsic limb muscles in dogs at walk, trot and gallop. J Exp Biol. 2012;215: 287–300. 10.1242/jeb.063230 22189773

[60] Ivanenko YP, Cappellini G, Dominici N, Poppele RE, Lacquaniti F. Coordination of locomotion with voluntary movements in humans. J Neurosci. 2005;25: 7238–7253. 10.1523/JNEUROSCI.1327-05.2005 16079406 PMC6725226

[61] Feeney LC, Lin C-F, Marcellin-Little DJ, Tate AR, Queen RM, Yu B. Validation of two-dimensional kinematic analysis of walk and sit-to-stand motions in dogs. Am J Vet Res. 2007;68: 277–282. 10.2460/ajvr.68.3.277 17331017

[62] Kim J, Rietdyk S, Breur GJ. Comparison of two-dimensional and three-dimensional systems for kinematic analysis of the sagittal motion of canine hind limbs during walking. Am J Vet Res. 2008;69: 1116–1122. 10.2460/ajvr.69.9.1116 18764680

[63] Corazza S, Gambaretto E, Mündermann L, Andriacchi TP. Automatic generation of a subject-specific model for accurate markerless motion capture and biomechanical applications. IEEE Trans Biomed Eng. 2010;57: 806–812. 10.1109/TBME.2008.2002103 19272951

[64] Muybridge E. Animals in Motion. Courier Dover Publications; 1957.

[65] Shapiro LJ, Raichlen DA. Lateral sequence walking in infant Papio cynocephalus: Implications for the evolution of diagonal sequence walking in primates. Am J Phys Anthropol. 2005;126: 205–213. 10.1002/ajpa.20049 15386221

[66] MacLellan MJ, Ivanenko YP, Cappellini G, Sylos Labini F, Lacquaniti F. Features of hand-foot crawling behavior in human adults. J Neurophysiol. 2012;107: 114–125. 10.1152/jn.00693.2011 21975454

[67] Fischer MS, Blickhan R. The tri-segmented limbs of therian mammals: kinematics, dynamics, and self-stabilization—a review. J Exp Zoolog A Comp Exp Biol. 2006;305A: 935–952. 10.1002/jez.a.333 17029268

[68] Bianchi L, Angelini D, Orani GP, Lacquaniti F. Kinematic coordination in human gait: relation to mechanical energy cost. J Neurophysiol. 1998;79: 2155–2170. 9535975 10.1152/jn.1998.79.4.2155

[69] Fisher NI, Lewis T, Embleton BJJ. Statistical Analysis of Spherical Data. Reprint edition. Cambridge; New York, NY, USA: Cambridge University Press; 1993.

[70] Leong P, Carlile S. Methods for spherical data analysis and visualization. J Neurosci Methods. 1998;80: 191–200. 9667392 10.1016/s0165-0270(97)00201-x

[71] Lee DD, Seung HS. Learning the parts of objects by non-negative matrix factorization. Nature. 1999;401: 788–791. 10.1038/44565 10548103

[72] Berens P. CircStat: A MATLAB Toolbox for Circular Statistics. J Stat Softw. 2009;31: 1–21.

[73] DeCamp CE. Kinetic and kinematic gait analysis and the assessment of lameness in the dog. Vet Clin North Am Small Anim Pract. 1997;27: 825–840. 9243783 10.1016/s0195-5616(97)50082-9

[74] Goldner B, Fuchs A, Nolte I, Schilling N. Kinematic adaptations to tripedal locomotion in dogs. Vet J. 2015;[in press]. 10.1016/j.tvjl.2015.03.003 25862392

[75] Ivanenko YP, Grasso R, Macellari V, Lacquaniti F. Control of foot trajectory in human locomotion: role of ground contact forces in simulated reduced gravity. J Neurophysiol. 2002;87: 3070–3089. 12037209 10.1152/jn.2002.87.6.3070

[76] Giszter SF, Hart CB, Silfies SP. Spinal cord modularity: evolution, development, and optimization and the possible relevance to low back pain in man. Exp Brain Res Exp Hirnforsch Expérimentation Cérébrale. 2010;200: 283–306. 10.1007/s00221-009-2016-x PMC2861904 19838690

[77] Dominici N, Ivanenko YP, Cappellini G, d’ Avella A, Mondì V, Cicchese M, et al Locomotor primitives in newborn babies and their development. Science. 2011;334: 997–999. 10.1126/science.1210617 22096202

[78] Dickinson MH, Lehmann FO, Sane SP. Wing rotation and the aerodynamic basis of insect flight. Science. 1999;284: 1954–1960. 10373107 10.1126/science.284.5422.1954

[79] Hsieh ST, Lauder GV. Running on water: Three-dimensional force generation by basilisk lizards. Proc Natl Acad Sci U S A. 2004;101: 16784–16788. 10.1073/pnas.0405736101 15550546 PMC534722

[80] Bush J, Hu D. Walking on water: Biolocomotion at the Interface. Annu Rev Fluid Mech. 2006;38: 339–369. 10.1146/annurev.fluid.38.050304.092157

[81] Bohn HF, Thornham DG, Federle W. Ants swimming in pitcher plants: kinematics of aquatic and terrestrial locomotion in Camponotus schmitzi. J Comp Physiol A. 2012;198: 465–476. 10.1007/s00359-012-0723-4 22526112

[82] Marsolais GS, McLean S, Derrick T, Conzemius MG. Kinematic analysis of the hind limb during swimming and walking in healthy dogs and dogs with surgically corrected cranial cruciate ligament rupture. J Am Vet Med Assoc. 2003;222: 739–743. 12675295 10.2460/javma.2003.222.739

[83] Fish FE, Dinenno NK. The dog paddle’: Stereotypic swimming gait pattern in different dog breeds Oxford Univ Press Inc; 2014 pp. E65–E65. 10.1002/ar.24396 32243718

[84] Niedźwiedzki G, Szrek P, Narkiewicz K, Narkiewicz M, Ahlberg PE. Tetrapod trackways from the early Middle Devonian period of Poland. Nature. 2010;463: 43–48. 10.1038/nature08623 20054388

[85] Tan U. Two families with quadrupedalism, mental retardation, no speech, and infantile hypotonia (Uner Tan Syndrome Type-II); a novel theory for the evolutionary emergence of human bipedalism. Evol Psychol Neurosci. 2014;8: 84 10.3389/fnins.2014.00084 PMC4001073 24795558

[86] Meyns P, Bruijn SM, Duysens J. The how and why of arm swing during human walking. Gait Posture. 2013;38: 555–562. 10.1016/j.gaitpost.2013.02.006 23489950

[87] Wannier T, Bastiaanse C, Colombo G, Dietz V. Arm to leg coordination in humans during walking, creeping and swimming activities. Exp Brain Res. 2001;141: 375–379. 11715082 10.1007/s002210100875

[88] Cheron G, Bouillot E, Dan B, Bengoetxea A, Draye JP, Lacquaniti F. Development of a kinematic coordination pattern in toddler locomotion: planar covariation. Exp Brain Res. 2001;137: 455–466. 11355390 10.1007/s002210000663

[89] Noble JW, Prentice SD. Intersegmental coordination while walking up inclined surfaces: age and ramp angle effects. Exp Brain Res. 2008;189: 249–255. 10.1007/s00221-008-1464-z 18584161

[90] Hallemans A, Aerts P. Effects of visual deprivation on intra-limb coordination during walking in children and adults. Exp Brain Res. 2009;198: 95–106. 10.1007/s00221-009-1937-8 19618172

[91] Maclellan MJ, McFadyen BJ. Segmental control for adaptive locomotor adjustments during obstacle clearance in healthy young adults. Exp Brain Res. 2010;202: 307–318. 10.1007/s00221-009-2133-6 20049421

[92] Ivanenko YP, d’ Avella A, Poppele RE, Lacquaniti F. On the origin of planar covariation of elevation angles during human locomotion. J Neurophysiol. 2008;99: 1890–1898. 10.1152/jn.01308.2007 18272871

[93] Dominici N, Ivanenko YP, Cappellini G, Zampagni ML, Lacquaniti F. Kinematic strategies in newly walking toddlers stepping over different support surfaces. J Neurophysiol. 2010;103: 1673–1684. 10.1152/jn.00945.2009 20089810

[94] Leurs F, Bengoetxea A, Cebolla AM, De Saedeleer C, Dan B, Cheron G. Planar covariation of elevation angles in prosthetic gait. Gait Posture. 2012;35: 647–652. 10.1016/j.gaitpost.2011.12.017 22257927

[95] Laroche D, Ornetti P, Thomas E, Ballay Y, Maillefert JF, Pozzo T. Kinematic adaptation of locomotor pattern in rheumatoid arthritis patients with forefoot impairment. Exp Brain Res. 2007;176: 85–97. 10.1007/s00221-006-0597-1 16915399

[96] Ijspeert AJ, Crespi A, Ryczko D, Cabelguen J-M. From Swimming to Walking with a Salamander Robot Driven by a Spinal Cord Model. Science. 2007;315: 1416–1420. 10.1126/science.1138353 17347441

[97] Revzen S, Guckenheimer JM. Estimating the phase of synchronized oscillators. Phys Rev E Stat Nonlin Soft Matter Phys. 2008;78: 051907 19113155 10.1103/PhysRevE.78.051907

[98] Dounskaia N. Control of human limb movements: the leading joint hypothesis and its practical applications. Exerc Sport Sci Rev. 2010;38: 201–208. 10.1097/JES.0b013e3181f45194 20871237 PMC2965031

[99] McCrea DA, Rybak IA. Organization of mammalian locomotor rhythm and pattern generation. Brain Res Rev. 2008;57: 134–146. 17936363 10.1016/j.brainresrev.2007.08.006 PMC2214837

[100] Hart CB, Giszter SF. A neural basis for motor primitives in the spinal cord. J Neurosci Off J Soc Neurosci. 2010;30: 1322–1336. 10.1523/JNEUROSCI.5894-08.2010 PMC6633785 20107059

[101] Prochazka A, Ellaway P. Sensory systems in the control of movement. Compr Physiol. 2012;2: 2615–2627. 10.1002/cphy.c100086 23720260

[102] Cheron G, Duvinage M, De Saedeleer C, Castermans T, Bengoetxea A, Petieau M, et al From spinal central pattern generators to cortical network: integrated BCI for walking rehabilitation. Neural Plast. 2012;2012: 375148 10.1155/2012/375148 22272380 PMC3261492

[103] Drew T, Kalaska J, Krouchev N. Muscle synergies during locomotion in the cat: a model for motor cortex control. J Physiol. 2008;586: 1239–1245. 10.1113/jphysiol.2007.146605 18202098 PMC2375657

[104] Armstrong DM. The supraspinal control of mammalian locomotion. J Physiol. 1988;405: 1–37. 3076600 10.1113/jphysiol.1988.sp017319 PMC1190962

[105] Pearson KG. Generating the walking gait: role of sensory feedback. Prog Brain Res. 2004;143: 123–129. 10.1016/S0079-6123(03)43012-4 14653157

[106] Miller S, Van Der Burg J, Van Der Meché F. Coordination of movements of the kindlimbs and forelimbs in different forms of locomotion in normal and decerebrate cats. Brain Res. 1975;91: 217–237. 1164672 10.1016/0006-8993(75)90544-2

[107] Yamaguchi T. Descending pathways eliciting forelimb stepping in the lateral funiculus: experimental studies with stimulation and lesion of the cervical cord in decerebrate cats. Brain Res. 1986;379: 125–136. 3742207 10.1016/0006-8993(86)90264-7

[108] Gerasimenko Y, Musienko P, Bogacheva I, Moshonkina T, Savochin A, Lavrov I, et al Propriospinal Bypass of the Serotonergic System That Can Facilitate Stepping. J Neurosci. 2009;29: 5681–5689. 10.1523/JNEUROSCI.6058-08.2009 19403834 PMC2940277

[109] Juvin L, Gal J-PL, Simmers J, Morin D. Cervicolumbar Coordination in Mammalian Quadrupedal Locomotion: Role of Spinal Thoracic Circuitry and Limb Sensory Inputs. J Neurosci. 2012;32: 953–965. 10.1523/JNEUROSCI.4640-11.2012 22262893 PMC6621141

[110] Beliez L, Barrière G, Bertrand SS, Cazalets J-R. Origin of Thoracic Spinal Network Activity during Locomotor-Like Activity in the Neonatal Rat. J Neurosci. 2015;35: 6117–6130. 10.1523/JNEUROSCI.4145-14.2015 25878284 PMC6605171

[111] Zelenin PV, Deliagina TG, Orlovsky GN, Karayannidou A, Dasgupta NM, Sirota MG, et al Contribution of Different Limb Controllers to Modulation of Motor Cortex Neurons during Locomotion. J Neurosci. 2011;31: 4636–4649. 10.1523/JNEUROSCI.6511-10.2011 21430163 PMC3073383

