# Supplementary material for: Planar Covariation of Hindlimb and Forelimb Elevation Angles during Terrestrial and Aquatic Locomotion of Dogs
Source: PLoS One. 2015 Jul 28;10(7):e0133936. doi: 10.1371/journal.pone.0133936 (PMC4517757; doi:10.1371/journal.pone.0133936)
Supplement: S1 Dataset — S1_Dataset.zip contains two folders: 1) Files in the 'Dogs Data' folder are the kinematic data in Matlab format (.mat) for each subject and each gait; 2) Files in the 'comparison' folder are the data used for the validation of the markerless motion capture system. (ZIP) [file pone.0133936.s001.zip › S1_DataSet/readme.pdf]

S1_DataSet.zip contains two folders

- Dogs Data: contains six subjectname.mat files.
- comparison:

1) Files in the 'Dogs Data' folder are all the subjects' data mat files: 'DogA.mat','DogG.mat','DogK.mat','DogM.mat','DogR.mat','DogS.mat'.
Each file is a Matlab file containing the subjects' data structures (named 'dogsdata') used in the analysis presented in the manuscript.
Each 'dogsdata' structure contains nested substructures, one per motion (walking, trotting, galloping, swimming).
These substructures contain the following fields: forelimb, hindlimb and timing, which lengths are equal to the number of trials.

Here after we will refer to:
N = number of total strides in the trial
M = number of total strides of contralateral limb strides in the trial
contralateral or contralat refers to the limb doesn't face the camera
T = total frame number for each trial

Forelimb substructure is organized with the following fields:
- fname: name of the file with the structure: subjectname_trialnumber_motion_frameratefps (fps = frame per second)
- cp (cycle parse): for each stride the frame relative to, respectively, touch down, push off, and touch down events is reported in each row.
- td (touch down): frames relative to touch down's events
- po (push off): frames relative to push off's events
- contralatcp: cycle parse of contralateral limb, data organized as described for the cp field
- contralattd: frames relative to touch down's events of the contralateral limb
- contralatpo: see po
- indselcp: selected strides used in the present analysis.
- contralatindselcp: see indselcp
- lead: logical vector of length N, lead(i) = 1 if the limb is trailing, lead(i) = 0 if it is leading, for i = 1,...,N. for symmetrical gaits lead(i) = 1 for each i = 1,...,N
- clead: logical vector of length M, see lead, relative to contralateral limb
- scapula: vector of length T, elevation angles of scapula segment
- upperarm: vector of length T, elevation angle of upperarm segment
- lowerarm: vector of length T, elevation angle of lowerarm segment
- hand: vector of length T, elevation angle of hand segment
- SVD: structure obtained from PCA analysis of upperarm, lowerarm and hand elevation angles, it contains the following fields:
    - U: eigenmatrix
    - D: eigenvalue matrix
    - X: data matrix centered
    - PV: eigenvector percent of variance
    - xxgrid: coordinates used to plot the covariance plane in matlab
    - z3: see xxgrid
- SVD2: same structure as SVD, in this case is from PCA analysis of scapula, upperarm, lowerarm
- gait: substructure containing the list of features used in the present analysis for each stride:
    - label: stride number
    - stride_time: in seconds
    - gaitstart: in seconds

- stance_time: in seconds
- swing_time: in seconds
- stance_perc: in percent of cycle
- swing_perc: in percent of cycle
- SCAPULA: scapula elevation angle interpolated at 100 points
- UPPERARM: upperarm elevation angle interpolated at 100 points
- LOWERARM: lowerarm elevation angle interpolated at 100 points
- HAND: lowerarm elevation angle interpolated at 100 points

- contralatgait: substructure containing the list of features used in the present analysis for each stride relative to the contralateral limb:
  - label, stride number
  - stride_time, in seconds
  - gaitstart, in seconds
  - stance_time, in seconds
  - swing_time, in seconds
  - stance_perc, in percent of cycle
  - swing_perc, in percent of cycle
- P, substructure containing the video coordinates of forelimb markers in meters
  - label: forelimbs' marker label
  - the other fields are the markers' coordinates

Hindlimb substructure is organized with the following fields: (in the following only the fields not included in the forelimb substructure are described)

- thigh, elevation angle of thigh segments
- shank, elevation angle of shank segments
- foot, elevation angle of foot segments
- toes, elevation angle of toes segments
- SVD: substructure obtained from PCA analysis of thigh, shank and foot elevation angles, it contains the same fields of forelimbs' SVD substructure
- gait: substructure containing list of features used in the present analysis for each stride relative to the hindlimb, it contains the same fields of forelimbs' gait substructure, exept for the following:
  - THIGH: thigh elevation angle interpolated at 100 points
  - SHANK: shank elevation angle interpolated at 100 points
  - FOOT: foot elevation angle interpolated at 100 points
- P: substructure with video coordinates of hindlimb markers in meters
  - label: hindlimbs' marker albel
  - the other fields are the markers' coordinates

Timing substructure is organized with the following fields:

- name:
- I (stride events Index): frame rate of td,po,td events
- tao: logical vector of length T, tao(i) = 1, in that frame the dog has no limb touching the ground, tao(i) = 0, the dog has at least one limb touching the ground, for i = 1,...,N
- PL: phase lag matrix

2) Files in the 'comparison' folder are the data used for validation of the markerless motion capture system. The data are the following

- DogS_fromSIMI, data obtained with the SIMI motion capture system
- DogS_fromvideo, data obtained with markerless method, from video recorded simultaneously with SIMI system.

In this folder the structures have the same fields of forelimb and hindlimb structures previously described.
